# Ultradian hydrocortisone replacement alters neuronal processing, emotional ambiguity, affect and fatigue in adrenal insufficiency: The PULSES trial

**DOI:** 10.1111/joim.13721

**Published:** 2023-10-19

**Authors:** Georgina Russell, Konstantinos Kalafatakis, Claire Durant, Nicola Marchant, Jamini Thakrar, Russell Thirard, Jade King, Jane Bowles, Thomas Upton, Ngoc Jade Thai, Jonathan C. W. Brooks, Aileen Wilson, Kirsty Phillips, Stuart Ferguson, Meryem Grabski, Chris A. Rogers, Theodoros Lampros, Sue Wilson, Catherine Harmer, Marcus Munafo, Stafford L. Lightman

**Affiliations:** ^1^ Laboratories of Integrative Neuroscience and Endocrinology Bristol Medical School University of Bristol Bristol UK; ^2^ University Hospital Bristol and Weston NHS Foundation Trust Bristol UK; ^3^ North Bristol NHS Trust Bristol UK; ^4^ Department of Informatics and Telecommunications, Human‐Computer Interaction Laboratory University of Ioannina Arta Greece; ^5^ Clinical Research and Imaging Centre University of Bristol Bristol UK; ^6^ Faculty of Medicine and Dentistry (Malta Campus) Queen Mary University of London Victoria Malta; ^7^ Department of Brain Sciences Faculty of Medicine Imperial College London London UK; ^8^ Bristol Trials Centre Bristol Medical School University of Bristol Bristol UK; ^9^ Neurosciences and Mental Health Liverpool Health Partners Liverpool UK; ^10^ School of Psychology University of East Anglia Norwich UK; ^11^ School of Medicine University of Tasmania Hobart Tasmania Australia; ^12^ Division of Psychology and Language Sciences UCL London UK; ^13^ Department of Psychiatry Oxford University and Oxford Health NHS Foundation Trust Oxford UK; ^14^ MRC Integrative Epidemiology Unit School of Psychological Science University of Bristol Bristol UK

**Keywords:** fMRI, glucocorticoid replacement therapy, primary adrenal insufficiency, ultradian

## Abstract

**Background:**

Primary adrenal insufficiency (PAI) mortality and morbidity remain unacceptably high, possibly arising as glucocorticoid replacement does not replicate natural physiology. A pulsatile subcutaneous pump can closely replicate cortisol's circadian and ultradian rhythm.

**Objectives:**

To assess the effect of pump therapy on quality of life, mood, functional neuroimaging, behavioural/cognitive responses, sleep and metabolism.

**Methods:**

A 6‐week randomised, crossover, double‐blinded and placebo‐controlled feasibility study of usual dose hydrocortisone in PAI administered as either pulsed subcutaneous or standard care in Bristol, United Kingdom (ISRCTN67193733). Participants were stratified by adrenal insufficiency type. All participants who received study drugs are included in the analysis. The primary outcome, the facial expression recognition task (FERT), occurred at week 6.

**Results:**

Between December 2014 and 2017, 22 participants were recruited – 20 completed both arms, and 21 were analysed. The pump was well‐tolerated. No change was seen in the FERT primary outcome; however, there were subjective improvements in fatigue and mood. Additionally, functional magnetic resonance imaging revealed differential neural processing to emotional cues and visual stimulation. Region of interest analysis identified the left amygdala and insula, key glucocorticoid‐sensitive regions involved in emotional ambiguity. FERT post hoc analysis confirmed this response. There were four serious adverse events (AE): three intercurrent illnesses requiring hospitalisation (1/3, 33.3% pump) and a planned procedure (1/1, 100% pump). There was a small number of expected AEs: infusion site bruising/itching (3/5, 60% pump), intercurrent illness requiring extra (3/7, 42% pump) and no extra (4/6, 66% pump) steroid.

**Conclusions:**

These findings support the administration of hormone therapy that mimics physiology.

## Graphical abstract

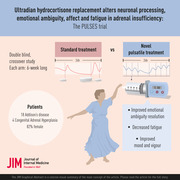

## Introduction

Primary adrenal insufficiency (PAI) is rare, with a prevalence of 100–200/million in the western world. In approximately 80% of cases, Addison's disease (AD) arises from autoimmune adrenal gland destruction. More rarely it can be caused by an enzyme defect in the cortisol biosynthetic pathway (congenital adrenal hyperplasia [CAH]). Secondary adrenal failure is more frequent, with a prevalence of 150–280/million, whilst tertiary adrenal insufficiency following withdrawal of chronic exogenous steroid treatment is a major global health problem [1, 2]. All these patients require long‐term glucocorticoid replacement, typically oral hydrocortisone given in divided dosages to mimic circadian variation, with a total daily dose (TDD) of 15–25 mg [3]. Despite presumed adequate replacement, patients describe significant morbidity, especially physical and mental fatigue, memory and concentration impairments, and affective disorders. This impacts quality of life (QoL) and social functioning [1, 3, 4, 5]. Patients also have increased metabolic risk and higher mortality from cardiovascular and infectious disease, cancer and unexplained death [3]. There have been many suggestions for these associations but unfortunately none convincingly causative.

Cortisol is the end‐product of the hypothalamic‐pituitary‐adrenal (HPA) axis. It is secreted in a circadian rhythm with an anticipatory awakening rise, secondary to changing amplitudes of discrete cortisol secretory pulses from a complex underlying ultradian rhythm. Ultradian rhythmicity emerges as a natural consequence of the feedforward and feedback interaction between the pituitary and adrenal cortex [5]. These hormonal oscillations produce transcriptional responses allowing tissue‐specific decoding of the ultradian signal in the brain, liver and immune system [6, 7]. Dysfunction of this complex neuroendocrine‐immune interface is associated with abnormalities of inflammatory mediators which may play a role in atopic/allergic disease, autoimmunity, obesity, depression and atherosclerosis. These abnormalities also affect the brain and metabolic pathways, modifying behavioural parameters, QoL, sleep and indices of metabolic and cardiovascular health [8].

Current replacement therapy not only misses cortisol's anticipatory rise but also lacks underlying ultradian rhythmicity. There are novel approaches to tackle circadian rhythmicity, including once‐daily and modified‐release oral preparations; these additionally do not address pulsatility [9, 10]. We developed a pulsatile subcutaneous infusion paradigm that mimics circadian and ultradian rhythmicity [11]. Here we report the PULSES trial, which aimed to assess the impact of ultradian hormone replacement on emotional processing, fatigue, working memory and metabolic status. Psychological and metabolic objectives were chosen to be representative of issues important to patients and clinicians.

## Materials and methods

### Study design

**Fig. 1:**
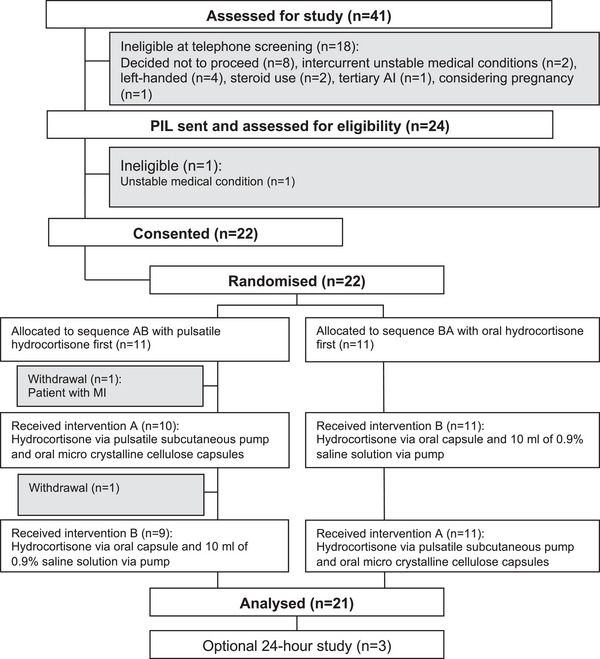
Trial profile. Local institutional, NHS Health Research Authority and Medicines Healthcare Regulatory Approval was obtained. The study was conducted in accordance with the principles of the Declarations of Helsinki and International Conference on Harmonisation E6 guidelines for Good Clinical Practice (GCP).

The PULSES trial was a 6‐week randomised, double‐blinded, crossover and placebo‐controlled study of usual dose hydrocortisone replacement therapy (range 20–30 mg) delivered as either pulsed subcutaneous pump treatment or standard three times daily oral at the University Hospital Bristol and Weston NHS Foundation Trust (UHBristol) in the United Kingdom. Local institutional and NHS Health Research Authority and Medicines Healthcare Regulatory approval was obtained. The study was sponsored by UHBristol (identification code: ME/2011/3709) and registered with ICRCTN (identification code: 67193733) and EudraCT (identification code: 2012‐001104‐37). The study was conducted in accordance with the principles of the Declarations of Helsinki and International Conference on Harmonisation E6 guidelines for Good Clinical Practice (GCP). A CONSORT flow diagram is provided in Fig. 1.

### Participants

Eligible participants were aged 18–64 years with a historic diagnosis of PAI secondary to AD or CAH, taking conventional glucocorticoid therapy (hydrocortisone or prednisolone) plus once daily fludrocortisone with a stable dose for at least 3 months. All subjects underwent a short synacthen test, of which one AD subject exhibited residual adrenal functioning with an increase in cortisol of 28 nmol/L. The remainders were unresponsive, with a peak cortisol of less than 50 nmol/L or undetectable. For CAH, all subjects had been diagnosed at birth or shortly afterwards, which were salt wasting, and females had virilising phenotypes. Any participants on prednisolone were converted to a bioequivalent dosage of hydrocortisone. Exclusion criteria included any episode of adrenal insufficiency in the last 6 months, significant unstable intercurrent illness, treatment within the last 3 months of another glucocorticoid‐requiring disease and cortisol metabolism‐interfering medications. A full list of inclusion and exclusion criteria are detailed in Table S1. Recruitment included consulting databases, patient support groups and local clinics/advertising campaigns. All participants provided written informed consent.

### Randomization and masking

Eligible participants were randomized to receive either pulsatile hydrocortisone and oral placebo first or pulsatile placebo and oral hydrocortisone by UHBristol Pharmacy Trials Unit using a web‐based application [12]. Randomisation was stratified by adrenal insufficiency type (AD vs. CAH). Participants, personnel and sponsor were masked to treatment assignments. In both treatment periods, participants were required to take the same daily regimen of over‐encapsulated tablets and have pump syringes with colourless liquid. Pseudoanonymized data from all outcomes were stored on the University of Bristol central server. Data were cleaned at an individual level and analysed at a group level without knowledge of treatment allocation.

### Procedures

All investigational medical products were prepared, stored and dispensed by UHBristol pharmacy. Participants either received hydrocortisone 5 mg over‐encapsulated in microcrystalline cellulose capsules and sodium chloride 0.9% 10 mL (placebo) in their pump, or they received placebo microcrystalline cellulose capsules and hydrocortisone sodium phosphate 100 mg in 10 mL sodium chloride 0.9% in their pump. Hydrocortisone sodium phosphate is a sterile aqueous solution for injection/infusion that is licenced for the treatment of hypoadrenalism. For the trial, it was administered off‐licence subcutaneously. The TDD of both oral and subcutaneous hydrocortisone was participants’ usual TDD (range 20–30 mg). Participants took capsules three times a day according to their usual routine, a standard 20 mg replacement regime being two tablets on awakening, one tablet at 12:00, and one at 16:00.

The Crono P subcutaneous portable infusion pump (CANE Applied Medical Technology Ltd) was pre‐programmed to deliver a high, medium and low sized pulse at a flow rate of 10 μL/s to replicate the circadian and ultradian rhythm of cortisol (Table S2) [12].

No change in dose was allowed except for intercurrent illness. Participants were advised regarding sick‐day rules and would increase their dose with study medications, seek medical assistance according to usual practice and notify the study team.

After randomization, participants underwent a 1‐week run‐in for pump training and baseline assessments. Participants received 6 weeks of blinded study treatment and were reviewed weekly. Participants texted/emailed post line change with residual volumes. At the end of 6 weeks, participants could stay on study treatment for up to one extra week to undergo a 24 h blood sampling study. All participants then completed a further week of assessments back on usual care. The assessments are presented in Table S3.

Participants completed a baseline assessment of mood using the Beck's Depression Inventory (BDI) at entry, and weekly anthropometric assessment of weight, blood pressure and body composition and an assessment of sleep using the Leeds Sleep Questionnaire.

Fasting blood samples (0800‐0900) were taken at baseline and at 6 weeks for metabolic biochemistry (total cholesterol, LDL, HDL/LDL ratio, insulin resistance homeostasis model assessment, triglycerides, HbA1c and osteocalcin). 24 h blood sampling occurred at UHBristol where 10‐min blood samples for cortisol and ACTH were taken via a human automated blood sampling system [13]. In CAH participants, 17‐OHP was also taken hourly. All samples were analysed at the department of clinical biochemistry, UHBristol.

Over 8 weeks (1‐week run‐in, 6‐weeks of treatment and 1‐week follow‐up), participants underwent Ecological Momentary Assessment (EMA) using an android phone and answered a fixed set of questions about self‐perceived reactivity and well‐being on a visual analogue scale (VAS) at multiple time points during each day [14] (Table S4) as well as a morning report of the Identity‐Consequence Fatigue Scale (ICFS) [15]. Participants were given the option of a 2‐week break during weeks 2–4. A total of 9334 assessments were collected.

QoL, mood and sleep questionnaires, and the N‐Back [14, 16] – a task of working memory – were completed at baseline and weeks 1 and 6. We used the Short Form 36 Health Survey (SF‐36) [17], Chalder fatigue scale [18], ICFS [15], the AD‐specific QoL score (AddiQoL) [4], positive and negative affect score (PANAS) [19] and the Pittsburgh Sleep Quality Index (PSQI) [20]. At week 6 as described in Kalafatakis et al., participants undertook a series of objective psychological tasks [14, 16]:

1. A series of emotion‐based paradigms, the ETB (P1 vital Emotional Test Battery), that capture biases in emotional processing [20]. This included: (i) the facial expression recognition task (FERT – facial expression interpretation), (ii) the Emotional Categorisation Task (ECAT – speed to respond to positive and negative self‐referent personality descriptors), (iii) the Faces Dot Probe Task (FDOT – attention to positive vs. negative stimuli) and (iv) the Emotional Recall Task (EREC – surprise free recall task examining incidental encoding of emotional stimuli).
2. Functional magnetic resonance imaging (fMRI): This included: (i) emotional stimulation processing – a series of male and female faces, each block consisting of images in one of three emotional categories: happy, sad and fearful, (ii) checkerboard visual stimulation and (iii) resting state session with a fixation cross.

All psychological tasks and questionnaires were chosen based on (1) symptoms important to patients from our patient and public involvement work including fatigue and brain fog, with its secondary effect on QoL and activities of daily living; and (2) the fact that glucocorticoids impact key brain regions involved in mood, anxiety and memory. This combination allowed assessment of symptoms (subjective and objective) and burden of disease.

### Outcomes

The primary outcome of the study was changed after discussion with the Data Monitoring Committee (DMC) from the difference in the ETB between treatment modalities to the difference in the FERT between treatment modalities, a single aspect of the ETB on which the study was powered. The FERT is an objective measure of emotional processing with excellent retest reliability [21] and would test highly glucocorticoid‐sensitive brain regions (our fMRI regions of interest) identified as important by our previous healthy volunteer work [16]. Secondary outcomes included the other aspects of the ETB and changes from baseline to week 6 in working memory, QoL, sleep, metabolic profile (fasting HbA1c, lipids, osteocalcin, insulin resistance, body composition and blood pressure), EMA and emotional processing using fMRI.

### Statistical analysis

Sample size calculation was based on the primary endpoint. Data from previous studies investigating pharmacological challenges in clinical populations [22] have indicated effect sizes for our measures of cognitive and emotional function equivalent to *d* ∼ 1.0. Anticipating a 20% drop‐out, a sample size of *N* = 20 was estimated to be more than sufficient to achieve an 80% power at an alpha level of 5%. A *p* value less than 0.05 was used to indicate statistical significance. The two periods and two treatments crossover design used the few participants available most effectively and adjusted for intra‐subject variability in treatment response.

Primary and secondary outcome analyses were performed according to a prespecified statistical analysis plan, which was finalized before any comparative analysis. To compensate for unbalanced data between the treatment groups, differences between the two administration modes, FERT, FDOT, ECAT and EREC, were compared using mixed linear model analysis for crossover designs adjusting for treatment (pulsatile vs. oral hydrocortisone administration), period (first vs. second treatment period) and sequence (pulsatile vs. oral hydrocortisone first) as fixed effects, and participant as a random effect. We used log transformation or other transformation to correct for non‐normally distributed data. Adjusted mean differences (MDs) and 95% confidence interval (CI) – and adjusted ratios of geometric means with 95% CI for logarithmically transformed data – are reported. Analysis of the EMA also involved performing a principal component analysis to reduce the nine VAS mood items to six (three positive and three negative) (Table S5). The analyses were conducted in STATA version 16.1 (StataCorp LP).

Descriptive analyses were performed on body composition (weight, resting metabolic rate and visceral fat) and metabolic biochemistry (total cholesterol, LDL, HDL/LDL ratio, insulin resistance homeostasis model assessment, triglycerides, HbA1c and osteocalcin) without any formal modelling.

For 24 h blood sampling, visual descriptions of the data and simple descriptive statistics were performed.

The primary analysis included all participants even if protocol violations had occurred (intention to treat). Safety was monitored throughout the study and included recording of adverse events (AEs). AEs were classified according to Common Terminology Criteria for AEs version 4.0. An independent DMC reviewed safety data broken down into allocation A/B and met annually to review study progress and AEs until study completion. There were no cases of unblinding. The study was audited at an institutional level in line with GCP guidelines.

In the fMRI experiment, to increase homogeneity we included only the data of right‐handed female AD (*N* = 13). Image processing and analysis were performed using FSL FMRIB software library v6.0 (Fig. S1) as previously described in our healthy volunteer studies [14, 16, 23].

## Results

### Characteristics of the cohort

Between December 2014 and December 2017, 41 individuals from the United Kingdom were screened for eligibility, and 22 were randomized – 11 to receive pulsatile hydrocortisone and oral placebo first followed by pulsatile placebo and oral hydrocortisone, and 11 to receive pulsatile placebo and oral hydrocortisone first followed by pulsatile hydrocortisone and oral placebo. One participant became ineligible prior to commencing the first study treatment due to an unstable new medical condition. One withdrew from the study during the washout period between the first and second study intervention; their data are included in the analysis. A total of 20 participants completed both study interventions (Fig. 1). Baseline characteristics of the modified intention‐to‐treat population are listed in Table 1. Participants were predominately female, and baseline characteristics were similar. Eighteen had AD and four CAH. The washout period between study interventions varied from 3 weeks up to 1 year.

**Table 1:** Baseline characteristics of the intention‐to‐treat (ITT) population.

	Pulsatile hydrocortisone first (*N* = 11)		Oral hydrocortisone first (*N* = 11)	
	*n*	%	*n*	%
Participants with AD	9/11	81.8%	9/11	81.8%
Age (AD participants) –median (IQR), years	54	(36.0, 55.0)	52	(33.0, 59.0)
Age (CAH participants) – median (IQR), years	40	(33.0, 46.0)	38	(34.0, 42.0)
AD duration – median (IQR), years	10	(7.0, 28.0)	8	(5.0, 13.0)
BMI – median (IQR), kg/m^2^	26	(22.7, 33.7)	26	(24.2, 30.9)
Female	8/11	72.7%	10/11	90.9%
Daily dose – median (IQR), mg	20	(20.0, 20.0)	25	(20.0, 25.0)
Beck's Depression Inventory – median (IQR)	6	(0, 22)	5	(0, 23)

Abbreviations: AD, Addison's disease; CAH, congenital adrenal hyperplasia; IQR, interquartile range.

### Emotional Test Battery responses

**Fig. 2:**
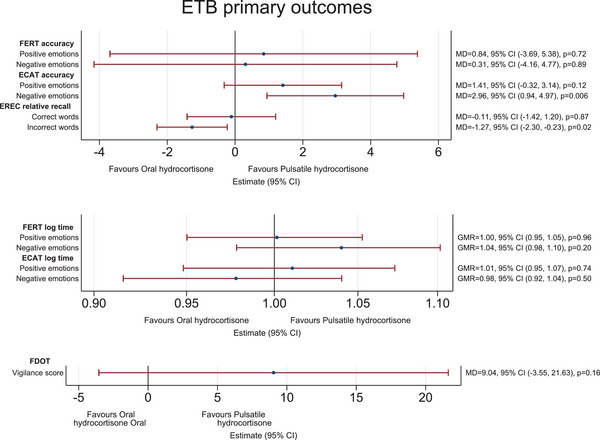
Emotional Test Battery treatment effect. Note: The top panel shows the difference in mean facial expression recognition task (FERT) and Emotional Categorisation Task (ECAT) accuracy, and EREC relative recall (blue circles) between the hydrocortisone administration groups (pulsatile minus oral). The middle panel shows the difference in FERT and ECAT time to respond on the logarithmic scale (blue circles) between the hydrocortisone administration groups (pulsatile over oral). The bottom panel shows the difference in mean Faces Dot Probe Task (FDOT) vigilance score (blue circle) between the hydrocortisone administration groups (pulsatile minus oral). The red line depicts the 95% confidence interval (CI). GMR, geometric mean ratio; MD, mean difference.

For the ETB FERT (Table 2, Fig. 2) primary outcome at 6 weeks, participants answered as quickly and with similar accuracy for both treatment periods. There was no treatment effect on accuracy (*p* = 0.72 and *p* = 0.89 for positive and negative faces, respectively). For the ECAT, participants on pulsatile classified more accurately positively (+1.5% on average) and especially negatively (+3% on average) valenced self‐referral personality descriptors (95% CI 0.94, 4.97, *p* = 0.006). There was a similar trend to identify positive emotions more accurately on pulsatile (MD [pulsatile minus oral] = 1.41 [−0.32,3.14], *p* = 0.12). For the EREC, participants irrespective of treatment were more likely to incorrectly recall more positive self‐referral descriptors from the ECAT. This was greatest on oral (MD = −1.27 [−2.30, −0.23], *p* = 0.02). Finally, the FDOT (attentional vigilance) showed no treatment difference (MD = −9.04 [−21.63, −3.55], *p* = 0.16).

**Table 2:** Primary (facial expression recognition task [FERT]) and secondary (Emotional Categorisation Task [ECAT], Faces Dot Probe Task [FDOT] and EREC) Emotional Test Battery outcomes; comparison between standard care and pulsatile pump treatment.

		Pulsatile pump hydrocortisone (*n* = 21)	Oral hydrocortisone (*n* = 20)^a^	Effect^b^ (95% CI)	*p*‐Value
FERT					
Accuracy – mean (SD), %	Positive	73.2 (4.6)	72.2 (7.5)	MD = 0.84 (−3.69,5.38)	0.72
	Negative	59.6 (9.5)	59.6 (10.9)	MD = 0.31 (−4.16,4.77)	0.89
Response time – median (IQR), ms	Positive	1469 (1351,1783)	1481 (1365,1672)	GMR = 1.00 (0.95,1.05)	0.96
	Negative	1676 (1478,2083)	1663 (1479,1881)	GMR = 1.04 (0.98,1.10)	0.20
ECAT					
Accuracy – median (IQR), %	Positive	100 (100,100)	98 (95,100)	MD = 1.41 (−0.32,3.14)	0.12
	Negative	100 (100,100)	98 (95,100)	MD = 2.96 (0.94,4.97)	0.006
Response time – median (IQR), ms	Positive	912 (880,1031)	945 (844,1031)	GMR = 1.01 (0.95,1.07)	0.74
	Negative	980 (898,1024)	1023 (909,1147)	GMR = 0.98 (0.92,1.04)	0.50
FDOT					
Vigilance score – mean (SD)	Masked	3.0 (25.1)	−5.8 (23.8)		
	Unmasked	8.3 (28.9)	−1 (29.5)	MD = −9.04 (−21.63, −3.55)	0.16
EREC					
Relative recall – mean (IQR) words	Correct	1.9 (2.3)	1.9 (2.4)	MD = −0.11 (−1.42,1.20)	0.87
	Incorrect	1.0 (1.4)	2.2 (2.3)	MD = −1.27 (−2.30, −0.23)	0.02

*Notes*: Data are median (IQR).

Abbreviations: CI, confidence interval; GMR, geometric mean ratio; IQR, interquartile range; MD, mean difference.

^a^ One participant withdrew after their first period.

^b^ Effect comparing oral and pulsatile hydrocortisone estimated in linear mixed models adjusted for period and sequence, and subgroup effects.

### Biochemical responses

Plasma ACTH and cortisol levels were measured in two AD and one CAH participant every 10 min for 24 h on each treatment. Visual data description (Fig. S2) indicated that on pulsatile cortisol levels rose at 03:00 with discrete pulses 3‐hourly, reaching a peak (range 315–779 nmol/L) between 06:50 and 10:10, gradually decreasing over the day to the nadir at approximately midnight (<50 nmol/L). On oral, cortisol levels did not rise until after awakening, with higher peak levels (552–1192 nmol/L) and similar circadian peak times (07:20–09:10) and nadir levels (<50 nmol/L). Despite receiving the same TDD, pulsatile AUC was greater (ratio 1.17–1.54). On pulsatile in AD, ACTH was low throughout the day. In CAH, ACTH levels started to rise in the awakening period and reached a peak of 113 pg/mL. On oral, large ACTH levels were seen commencing in the overnight period, reaching a peak level of 124–193 pg/mL, occurring between 07:00 and 09:10. AUC for pulsatile was smaller (ratio 0.17–0.95). 17‐OHP levels were also lower on pulsatile (123 vs. 388 pg/mL), as was AUC (ratio 0.6).

### fMRI

**Fig. 3:**
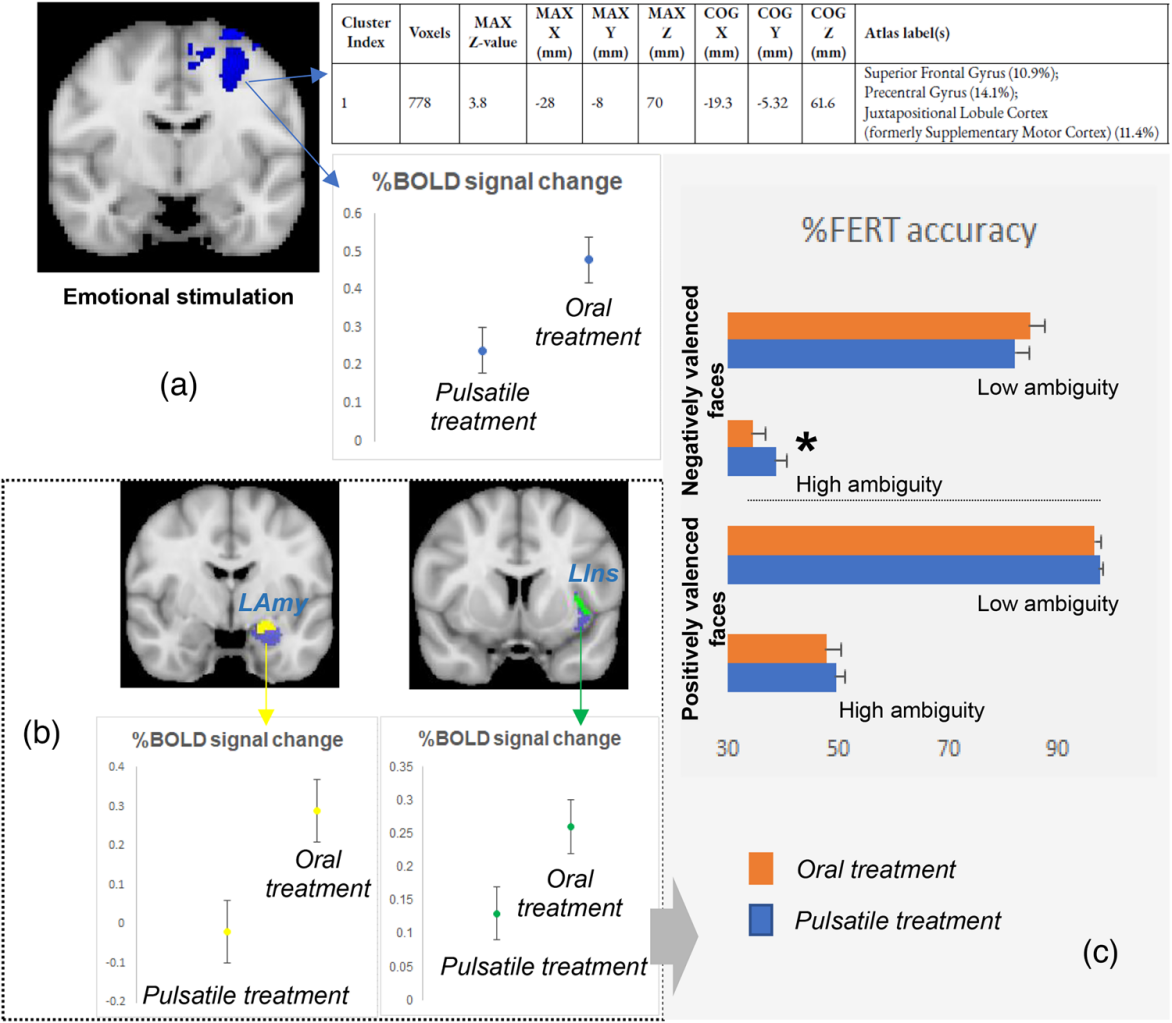
(a) Neural processing differences between the oral and pulsatile hydrocortisone replacement groups, under the same conditions of emotional stimulation (emotionally valenced face presentation). Whole‐brain analysis revealed a cluster of brain regions (containing 778 voxels, highlighted in blue, coronal view), corresponding to parts of the left superior frontal gyrus, precentral gyrus, supplementary motor cortex, anterior cingulate and paracingulate (based on the Harvard‐Oxford cortical structural atlas), showing a reduced (compared to baseline) per cent blood oxygen level dependent (BOLD) signal response to emotional stimulation in the pulsatile treatment group compared to the oral one (p < 0.0001). (b) Region of interest (ROI) analysis revealed similar, between‐treatment group differences in the per cent BOLD signal responses to emotional stimulation for parts (highlighted in yellow and green, respectively, coronal view) of the left amygdala (Lamy, blue) and left insula (LIns, blue). (c) Post hoc analysis of variance showed a comparatively reduced accuracy of the participants in the oral treatment to correctly identify highly ambiguous, negatively valenced faces (*). Data presented as group mean ± SD. FERT, facial expression recognition task; MAX z‐value, the maximum z‐statistic contained in the cluster; MAX X/Y/Z (mm), the location of the voxel with the maximum z‐statistic, given as X/Y/Z coordinate values in MNI152 standard space (mm); COG X/Y/Z (mm), the location of the centre of gravity for the cluster (i.e. a weighted average of the coordinates by the intensities within the cluster).

Whole‐brain fMRI analysis identified that a region of the left‐sided cerebral cortex (including superior and middle frontal gyrus, precentral gyrus, supplementary motor cortex, anterior cingulate and paracingulate gyri) (cluster size: 778 voxels; peak voxel MNI coordinates: *x* = −28, *y* = −8, *z* = 70; max *z*‐value = 3.8) showed differential neural processing in response to the fearful emotional stimulation depending on replacement mode (Fig. 3a). Region of interest (ROI) analysis revealed a similar, treatment mode‐dependent differential response of the left amygdala and insula to fear (Fig. 3b): Oral produced increased % blood oxygen level dependent (BOLD) signal changes compared to pulsatile.

Given these findings and the involvement of the amygdala and insula in processing emotional ambiguity [24], we performed a post hoc analysis of the ETB FERT data set, stratifying by treatment mode (oral vs. pulsatile), emotional face valence (positive vs. negative) and emotional ambiguity level (high ambiguity – facial emotion intensity 10%–50% – or low ambiguity – facial emotion intensity 60%–100%). Although this post hoc analysis did not reveal any interaction for positively valenced faces, it did show a significant interaction between the treatment mode and the level of emotional ambiguity for negatively valenced faces (*F*[1, 19] = 10.321, *p* = 0.005, *ω*2 = 0.31). Participants receiving pulsatile recognised more accurately negative cues of high ambiguity (*F*[1, 19] = 13.005, *p* = 0.002, *ω*2 = 0.36) (Fig. 3c), with no difference for low ambiguous, negatively valenced faces. Moreover, on pulsatile, participants were faster to recognise positively valenced faces and slower to recognise negatively valenced faces (*F*[1, 19] = 6.135, *p* = 0.023, *ω*2 = 0.20).

**Fig. 4:**
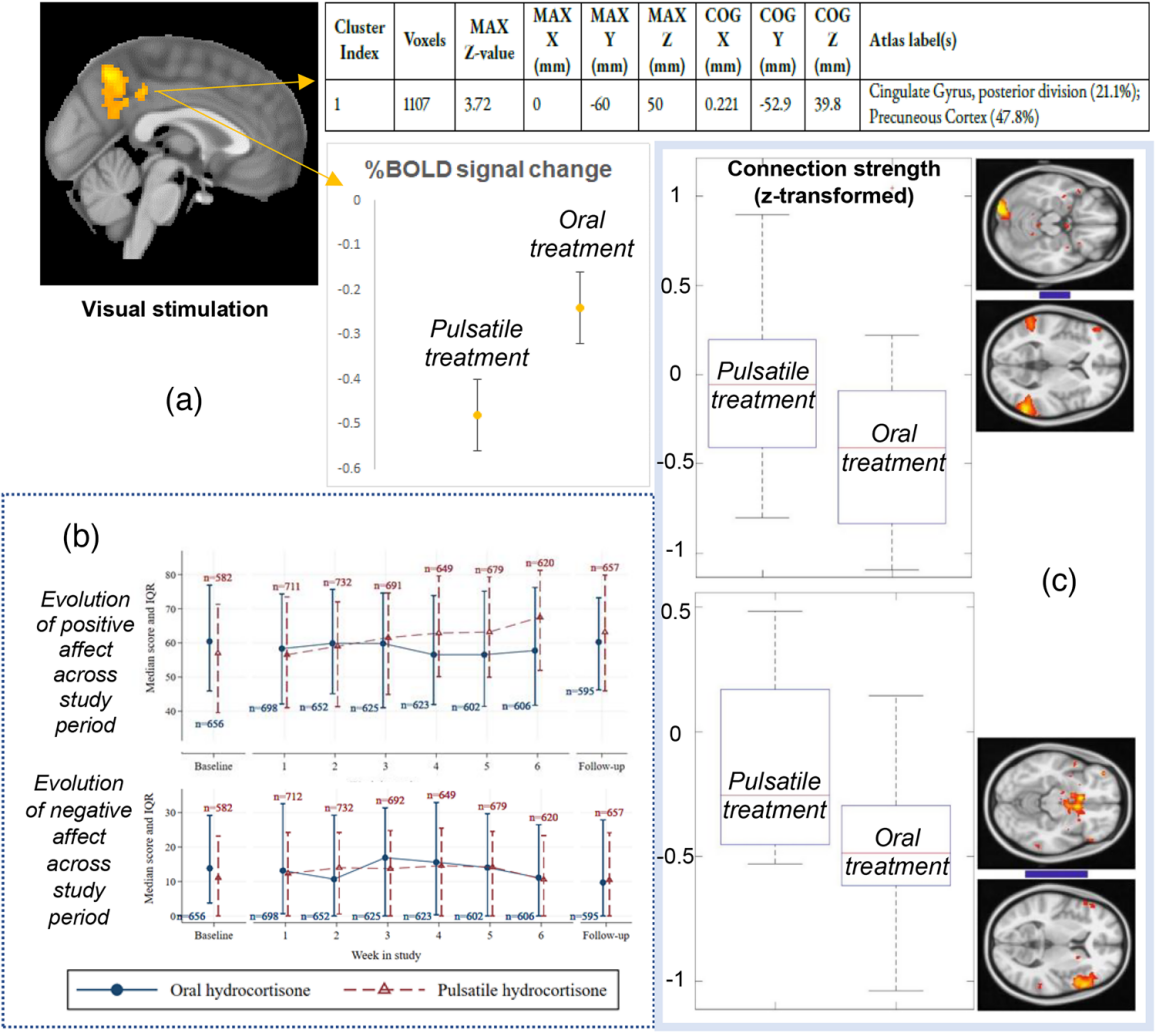
(a) Neural processing differences between the oral and pulsatile hydrocortisone replacement groups, under the same conditions of visual stimulation (flashing checkerboard). Whole‐brain analysis revealed a cluster of brain regions (containing 1107 voxels, highlighted in orange–yellow, sagittal view), corresponding to parts of the posterior cingulate and the precuneous (based on the Harvard‐Oxford cortical structural atlas), which show a reduced (compared to baseline) per cent blood oxygen level dependent (BOLD) signal response to visual stimulation in the pulsatile treatment group compared to the oral one (p < 0.0001). (b) Evolution of positive and negative affect index scores throughout the study period (at baseline, during the 6‐week study treatment period and at follow‐up, after the end of each treatment arm) for participants receiving either the oral or the pulsatile hydrocortisone replacement therapy. (c) The boxplots summarize the distribution of the correlation values (connection strengths) in the two groups – pulsatile and oral treatment – for a particular node‐pair, either the middle temporal gyri with the right occipital fusiform gyrus (upper pair of thumbnails) or the left inferior frontal gyrus with the subcallosal cortex (lower pair of thumbnails). The thickness of the blue‐coloured bar joining each pair of thumbnails indicates the strength of the overall group‐average connection, whereas the blue colour itself indicates that this connection is negative (i.e. the nodes anti‐correlate to each other on average). This anti‐correlation is comparatively stronger in the oral treatment group. Data presented as mean ± SD. IQR, interquartile range (range between 25th and 75th percentile of the data), MAX z‐value, the maximum z‐statistic contained in the cluster, MAX X/Y/Z (mm), the location of the voxel with the maximum z‐statistic, given as X/Y/Z coordinate values in MNI152 standard space (mm); COG X/Y/Z (mm), the location of the centre of gravity for the cluster (i.e. a weighted average of the coordinates by the intensities within the cluster).

Under checkerboard visual stimulation, whole‐brain analysis determined significant parts of the posterior cingulate cortex, and precuneous, principal nodes of the default mode network (DMN) (cluster size: 1107 voxels; peak voxel MNI coordinates: *x* = 0, *y* = −60, *z* = 50; max *z*‐value = 3.7) showed differential processing depending on replacement mode. Oral showed increased BOLD responses in these DMN nodes compared to pulsatile (Fig. 4a).

### Effects on QoL, mood, fatigue, working memory, sleep and metabolism

The EMA on the VAS mood revealed an improvement in positive affect index with pulsatile. Pulsatile versus oral MD = −1.70 (95% CI −3.22, −0.31, *p* = 0.03) on day 1 and increased to MD = 4.61 (95% CI 3.32, 5.91, *p* < 0.001) during the last treatment week (Fig. 4b, Table S6). The negative affect index score was lower with pulsatile (MD = −2.06, 95% CI [−2.58, −1.54], *p* < 0.001), and treatment difference did not change significantly over time. For the ICFS morning report, the difference in reported feelings of fatigue, vigour and energy between pulsatile and oral changed over time, with the difference being greatest during the last week of treatment (Table S7). Concentration (MD = −1.44, 95% CI [−2.74, −0.15], *p* < 0.05) and daily activities (MD 2.29, 95% CI [0.83, 3.75], *p* < 0.005) were lesser impacted with pulsatile.

These treatment mode‐dependent variations in context‐free cognition and emotion measures (concentration, liveliness, vigour, fatigue and positive affect) were not linked to significant changes in brain functional connectivity of resting state networks after comparative modelling. Compared to pulsatile, z‐transformed connection strength between the subcallosal cortex and left inferior frontal gyrus (min./Q1/median/Q3/max. pulsatile are −0.5/−0.45/−0.25/0.15/0.5 and oral are −1/−0.6/−0.5/−0.25/0.15) or between the middle temporal gyri with the right occipital fusiform gyrus (min./Q1/median/Q3/max. pulsatile are −0.75/−0.45/0/0.2/0.95 and oral are −1.1/−0.75/−0.45/−0.1/0.22) was weaker when participants received oral, though not significantly (*p* = 0.59 and *p* = 0.80, respectively) (Fig. 4c).

**Fig. 5:**
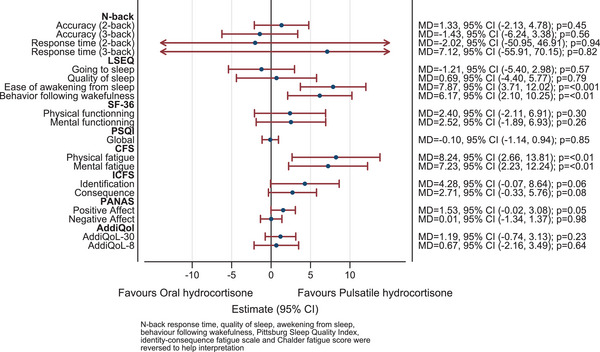
Secondary outcome measures treatment effects. Note: The figure shows the difference in mean response for the secondary outcomes (blue circles) between the hydrocortisone administration groups (pulsatile over oral). The red line depicts the 95% confidence interval (CI). N‐Back response time, quality of sleep, awakening from sleep, behaviour following wakefulness from the Leeds Sleep questionnaire (LSEQ), Pittsburgh Sleep Quality Index (PSQI), Identity‐Consequence Fatigue Scale (ICFS), Negative Affect Score and Chalder fatigue score (CFS) were reversed to help interpretation. MD, mean difference.

The N‐Back test did not differ. For sleep, the PSQI was greater than 5, indicating significant sleep disturbance on both modes but with less disruption on oral at week 1 (MD = 1.51, 95% CI [0.51, 2.52], *p* = 0.01). On pulsatile, sleep returned to baseline after 5 weeks (MD = 0.04, 95% CI [−0.96, 1.04], *p* = 0.94). The LSEQ detected no difference in going to and quality of sleep. Ease of awakening and behaviour following wakefulness was better with pulsatile (MD = −7.87, 95% CI [−12.02, −3.71], *p* ≤ 0.001 and MD = −6.17, 95% CI [−10.25, −2.10], *p* ≤ 0.01, respectively). Subjective measures of fatigue showed improvement in mental and physical fatigue (MD = −7.22, 95% CI [−12.10, 2.33], and MD = −8.22, 95% CI [−13.66, −2.78], *p* < 0.01, respectively) as well as lesser identification (MD = −4.28, 95% CI [−8.60, 0.04], *p* = 0.05) and consequence of fatigue (MD = −2.71, 95% CI [−5.69,0.27], *p* = 0.08). There was no change in the AddiQoL, SF36 physical and mental components, but greater positive affect on pulsatile (MD = 1.53, 95% CI [−0.02, 3.08], *p* = 0.05) was detected on the PANAS (Fig. 5, Figs. S3–S9). No change was seen in body composition (weight, resting metabolic rate and visceral fat) and metabolic biochemistry (total cholesterol, LDL, HDL/LDL ratio, insulin resistance homeostasis model assessment, triglycerides, HbA1c and osteocalcin) (Figs. S10 and S11).

### Adverse events

Pulsatile treatment was well tolerated (Table 3). The most common AE was extra steroid‐requiring intercurrent illness (3/7, 42% pulsatile). No participant discontinued treatment due to pump‐related AE. There were four serious AEs (SAE). Two SAEs were for hospital admission of less than 24 h that required use of intramuscular hydrocortisone for intercurrent illness, one on each treatment mode. There was one hospital admission for 2 days requiring intravenous hydrocortisone for intercurrent illness on oral, and a planned hospital admission for a pre‐existing condition on pulsatile.

**Table 3:** Adverse events and protocol deviations.

	Pulsatile hydrocortisone (*n* = 21)		Oral hydrocortisone (*n* = 20)	
	Events/participants	%	Events/participants	%
**Serious adverse events**				
Hospital admission <24 h	1/2	50.0	1/2	50
Hospital admission >24 h	0/1	0.0	1/1	100
Planned investigation	1/1	100	0/1	0
**Total**	**2/4**	**50.0**	**2/4**	**50**
**Adverse events**				
Bruising and itchiness at injection site	3/5	60.0	2/5	40
Fatigue or sleep issues	3/3	100	0/3	0
Intercurrent illness (requiring extra steroids)	3/7	42.9	4/7	57.1
Intercurrent illness (no extra steroid)	4/6	66.7	2/6	33.3
Other	1/2	50.0	1/2	50
**Total**	**14/23**	**60.9**	**9/23**	**39.1**
**Protocol deviations**				
Intercurrent illness (no extra steroid)	0/1	0.0	1/1	100
Line leakage	3/10	30.0	7/10	70
Technical issues with pump	1/2	50.0	1/2	50
User error	4/11	36.4	7/11	63.6
**Total**	**8/24**	**33.3**	**16/24**	**66.7**

## Discussion

In the PULSES randomised trial, administration of usual dose hydrocortisone delivered subcutaneously via a pump provided safe circadian and ultradian cortisol replacement. Pulsatile hydrocortisone altered both neural dynamics and behavioural responses related to emotional processing, visual stimulation and resting conditions, improved physical and mental fatigue, and reduced fatigue identification and consequence. Irrespective of treatment, participants had significant sleep disturbances, and treatment modality had no effect on sleep quality, although improved behaviour post awakening and ease of awakening was seen on pulsatile therapy. Pulsatility also improved positive mood. There was no effect on either working memory or metabolic parameters over the 6‐week trial.

Besides reduction in dosage, cortisol replacement has remained unchanged for many decades. It is widely recognised that current replacement therapy is unphysiological due to its lack of pre‐awakening surge, ultradian rhythmicity and post dose supraphysiological peaks [8]. Adrenally suppressed healthy volunteer data have shown over the short term (5 days) that our regime of pulsatile therapy closely mimics normal cortisol physiology and maintains ACTH within physiological levels [11]. In PULSES, 24 h blood sampling confirmed that pulsatile cortisol replacement replicated a circadian and ultradian rhythm. It is of particular interest that in AD subjects on pulsatile infusion, ACTH remained in the normal range overnight and remained at this level, suggesting appropriate endogenous HPA feedback. In the male CAH, levels were within target to two times the upper limit of normal (ULN). In contrast, oral replacement was associated with ACTH levels during the awakening phase two to three times ULN before falling back to normal (CAH) or suppressed (AD). In addition, for CAH, 17‐OHP levels – although still elevated – returned to close to normality in the afternoon and early evening and peaked at 100 nmol/L awakening, whilst oral therapy resulted in a similar daytime pattern, but peaked at almost 400 nmol/L – over three times pulsatile replacement.

HPA dysfunction is well recognized to be associated with depression, as well as poor sleep, well‐being, mood and cognition [24]. In PAI, affective disorders are higher, even in comparison to other chronic illnesses [25], and 40%–60% of patients need to make occupational or social changes [26]. In PULSES, we tested whether these issues are linked with mood and may arise, at least in part, to unphysiological replacement [27]. We chose to blind our study and utilize a mixture of subjective and objective psychological measures as study duration was short. The subjective questionnaires documented consistently a more positive affect on pulsatile treatment. After week 3, the EMA similarly recorded a gradual improvement in positive affect. However, the objective ETB results were surprising: Our previous, healthy volunteer work showed that pulsatile caused the ETB to exhibit a globalized shift towards positive affect, with attention towards positive emotions and misclassification of negative emotions as positive [16]. In PULSES, no objective treatment effect was seen in the primary outcome of the ETB FERT. In other ETB aspects, the ECAT exhibited greater accuracy in the classification of emotional responses on pulsatile, being particularly challenging for negative words as more ambiguous. The P1 vital ETB comprises five computerized tasks designed to assess human cognition and emotional processing. It has good test–retest reliability and can be used in cross‐over designs [21]. Our subjects, however, were not depressed. The BDI confirmed normal ups and downs of everyday life; indeed, the ETB showed that our participants consistently had a positive emotional bias.

The subjective mood responses in our subjects were associated with altered functional neuroimaging responses to external stimulation, with the different temporal patterns of plasma cortisol impacting brain areas important for emotional encoding, such as the amygdala, insula and frontal cortical regions. These changes were associated with altered accuracy in the identification of negatively valenced faces under conditions of emotional ambiguity. In other words, when it became more challenging under fearful conditions, participants were able to correctly classify subtle emotional cues.

Unlike our study in healthy volunteers – in which performance of working memory on the N‐Back improved on pulsatile – no changes were seen. This is akin to previous studies [28, 29] in which no difference was seen in comparison to matched controls for measurements of executive function – concentration and working, verbal and autobiographical memory [29] – despite self‐reported difficulties with executive functioning [28]. Possible explanations include that a 6‐week study does not provide enough time for central nervous system remodelling. Indeed, oral glucocorticoid replacement therapy in both CAH and AD has been shown over the longer term to be associated with an approximately 4% reduction in brain volumes, directly related to dose size [30].

The brain's ability to maintain optimal cognitive functioning is modulated by alterations in sleep and fatigue, with REM sleep being particularly important for emotional regulation [8, 31]. For sleep, all participants had a PSQI of greater than five, indicating significant sleep disturbances irrespective of treatment modality. Globally, pulsatile replacement was associated with greater sleep disturbance. This appeared to be greatest at 2 weeks and returned to baseline by week 6. This may have arisen as participants’ rhythm was disrupted and settled with time. We do not know whether sleep quality may have continued to improve with a longer duration of treatment. Indeed, studies have shown variable effects on slow wave sleep and reduced REM latency in PAI, all of which potentially impact cognition and emotion [31]. The reported disruption had no impact on perceived quality or ease of falling asleep. Most striking, on the other hand, was the improved ease of awakening and behaviour following awakening in the pulsatile infusion arm, presumably at least in part related to the presence of a pre‐awakening cortisol surge.

With regards to fatigue, patients consistently report impaired QoL, especially early morning mental and physical fatigue [3]. This increased morbidity has encouraged improved circadian hormone replacement with modified release oral glucocorticoid preparations (Chronocort, Plenadren) [9, 10] and circadian pumps [32]. Unfortunately, these have produced variable QoL findings. Open‐label and investigator‐double blinded trials have shown improvement [10], whereas double blinded and longer‐term studies show little or no change [27, 32]. In PULSES, patients consistently reported less physical and mental fatigue, and reduced consequence from it.

The strengths of PULSES include the randomised cross‐over design, strict inclusion criteria, blinding and run‐in and washout periods. In addition, this study utilised a combination of subjective and objective psychological measures. Previous studies have described difficulties with retention in blinded cross‐over trials. We had a rigorous protocol; consequently, self‐selecting highly motivated participants were recruited, which could impact upon fatigue, mood scores and the generalizability of our findings to the wider population. We also had a mix of CAH and AD, with different development time windows. CAH will have had abnormal glucocorticoid dynamics even in utero, whilst with AD, this effect occurs as a secondary event.

A further limitation is hydrocortisone dose. The decision was made to leave the TDD unchanged, only altering presentation dynamics. Although the administered doses were identical, the pulsatile AUC of cortisol was greater presumed secondary to delivery mode. Additionally, if as predicited pulsatile hydrocortisone is more bioactive because of effective gene responses due to gene pulsing, this might allow a dose reduction.

It is therefore clear from this discussion, as well as from previously published literature, that standardised assessment tools do not successfully quantify the symptoms/issues faced in PAI. The choice of primary outcome is therefore challenging, particularly as this needs to be objective. Our work suggests early changes are seen in amygdala activity; therefore, we would suggest a novel Facial Expression Ambiguity Resolution Task, precisely testing this key amygdala effect. With regards to the ideal characteristics of the cortisol infusion, we provide evidence that pulsatility is an important key component for improved responses. In future studies, the pattern and dose of the cortisol infusions can be further improved, allowing individual fine‐tuning. This should be easier now with new methodologies such as ambulatory microdialysis, which can provide details of cortisol responses [33]. Finally, longer duration studies can now be designed, allowing sufficient time to account for central remodelling.

## Conclusions

This is the first study to translate in vitro and in vivo animal studies, demonstrating the importance of cortisol pulsatility in a hormone‐deficient group of human patients and comparing this with previously recognised optimum therapy. We showed pulsatile ultradian hormone replacement improved affect, mental and physical fatigue and morning energy levels. Whole‐brain fMRI revealed differential neural processing to emotional cues and visual stimulation. ROI analysis confirmed changes in the left amygdala and insula. As these areas are involved in emotional ambiguity, post hoc analysis examining ambiguity of facial expression recognition confirmed this behavioural response. This has not only confirmed the relevance of previously performed animal studies to human patients but also provides an important guide to future therapeutics, suggesting that the pattern of hormone replacement is critical for normal cognition under non‐stressful conditions. This has wider‐reaching implications for the critically difficult aspect of glucocorticoid therapeutics – that is balancing the desired effects with unwanted side‐effects. Even short‐duration therapy at relatively low doses of glucocorticoids can cause neuropsychological side‐effects. The PULSES study has shown that the pattern of cortisol presentation, even at an assumed physiological dose, influences neuronal processes – suggesting that dosage is not the only variable that is important, but that we need to recapitulate normal ultradian physiology to truly minimise side‐effects.

## Author contributions

*Conceptualization; funding acquisition; investigation; fMRI formal analysis; fMRI data curation; formal analysis; data curation; project administration; writing – original draft; writing – reviewing and editing*: Georgina Russell. *Investigation; fMRI formal analysis; fMRI data curation; formal analysis; data curation; project administration; visualisation; writing – reviewing and editing*: Konstantinos Kalafatakis. *Investigation; writing – reviewing and editing*: Thomas Upton, Claire Durant, Jane Bowles. *Investigation; fMRI formal analysis; fMRI data curation; writing – reviewing and editing*: Jamini Thakrar, Jonathan C. W. Brooks, Ngoc Jade Thai. *Investigation; project administration; writing – reviewing and editing*: Nicola Marchant, Jade King. *Investigation; fMRI data curation; writing – reviewing and editing*: Aileen Wilson. *fMRI formal analysis; fMRI data curation; writing – reviewing and editing*: Theodoros Lampros. *Biochemistry resources; investigation; writing – reviewing and editing*: Kirsty Phillips. *Psychological conceptualization; psychological resources; psychological software; writing – reviewing and editing*: Catherine Harmer. *Psychological conceptualization; psychological resources; psychological software; EMA software; EMA resources; writing – reviewing and editing*: Marcus Munafo. *EMA software; EMA resources; writing – reviewing and editing*: Stuart Ferguson. *Formal analysis; data curation; project administration; visualisation; writing – original draft; writing – reviewing and editing*: Russell Thirard. *Formal analysis; data curation; project administration; writing – reviewing and editing*: Chris A. Rogers. *Formal analysis; data curation; writing – reviewing and editing*: Meryem Grabski, Sue Wilson. Conceptualization; funding acquisition; writing – reviewing and editing: Stafford L. Lightman. *Investigation; writing – reviewing and editing*: Thomas Upton.

## Conflict of interest statement

The authors declare that there are no conflicts of interest.

## Funding information

Medical Research Council, DPFS grant, MR/J012548/1 and the Above and Beyond Charities UHBristol, 04/2014‐15; Oxford Health NIHR Biomedical Research Centre

## Supporting information

Outline of the key methodological steps for the whole‐brain analysis of the functional brain images from the emotional stimulation experiment.

3 participants' 24‐hour blood profiles for cortisol, ACTH and 17‐OHP (CAH only). Foot note: Participants 1 and 2 were female AD and participant 3 was a male CAH. Red being pulsatile treatment and blue oral.

Mean score of the Pittsburg Sleep Quality Index (PSQI) at baseline, 1 week and 5 weeks.

Mean score of the weekly Leeds Sleep Evaluation Questionnaire. Red open triangle being pulsatile and blue closed circle oral hydrocortisone treatment.

Mean score of the Chalder Fatigue Score at baseline, week 1 and 5.

Mean score of the Identity Consequence Fatigue Scale (ICFS) at baseline, week 1 and 5.

Mean score of the Positive Affect Negative Affect Score (PANAS) at baseline, week 1 and 5.

Mean score of the Addison's Disease Quality of Life Scale (AddiQol‐30) at baseline, week 1 and 5.

Mean score of the Short Form 36 (SF36) at baseline, week 1 and 5.

Blood measures of total cholesterol, low density lipids and osteocalcin Blood measures of HDL/LDL metabolic ratio, triglycerides, HbA1c and Insulin resistance.

Body composition and resting metabolic rate.

Inclusion and exclusion criteria
Example of a total daily dose of 20mg delivered via the subcutaneous pump
Schematic diagram of tasks performed during each period of the study ETB (Emotional Test Battery), QoL (Quality of life questionnaires), EMA (Ecological Momentary Assessment), PANAS (Positive and Negative Affect Score), BDI (Beck's Depression Inventory), fMRI (functional Magnetic Resonance imaging)
EMA Mood VAS used in morning and evening reports and random prompts. The VAS‐assessment included 10 items: ‘energetic’, ‘alert’, ‘enthusiastic’, ‘happy’, ‘irritable’, ‘sad’, ‘stressed’, ‘unmotivated’,‘upset’ and ‘couch potato’. The latter item was excluded as it has not been validated by other studies. Items were scored on a visual analogue scale (0‐100).
Supplementary Table 5 Rotated factor loadings from the constrained two‐factor factor analysis fobrkr the 9 mood items

## Data Availability

The data that support the findings of this study are available on request from the corresponding author. The data are not publicly available due to privacy or ethical restrictions.
