# Supplementary figures and images for: Ultradian hydrocortisone replacement alters neuronal processing, emotional ambiguity, affect and fatigue in adrenal insufficiency: The PULSES trial
Source: J Intern Med. 2023 Oct 19;295(1):51–67. doi: 10.1111/joim.13721 (PMC10952319; doi:10.1111/joim.13721)

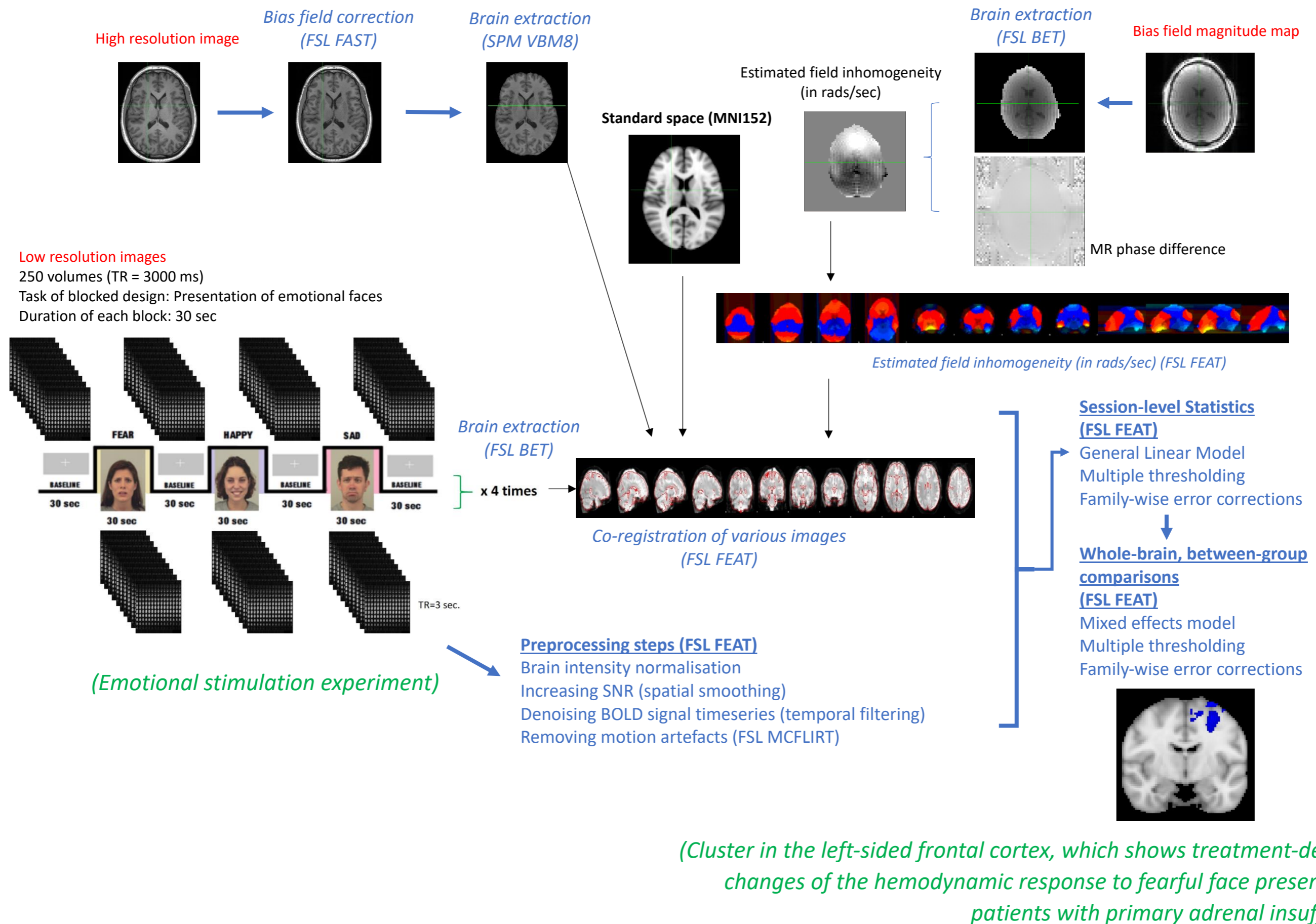

Supplement: Supplementary file 1 — Outline of the key methodological steps for the whole‐brain analysis of the functional brain images from the emotional stimulation experiment. [file JOIM-295-51-s012.pdf]

# PSQI global score

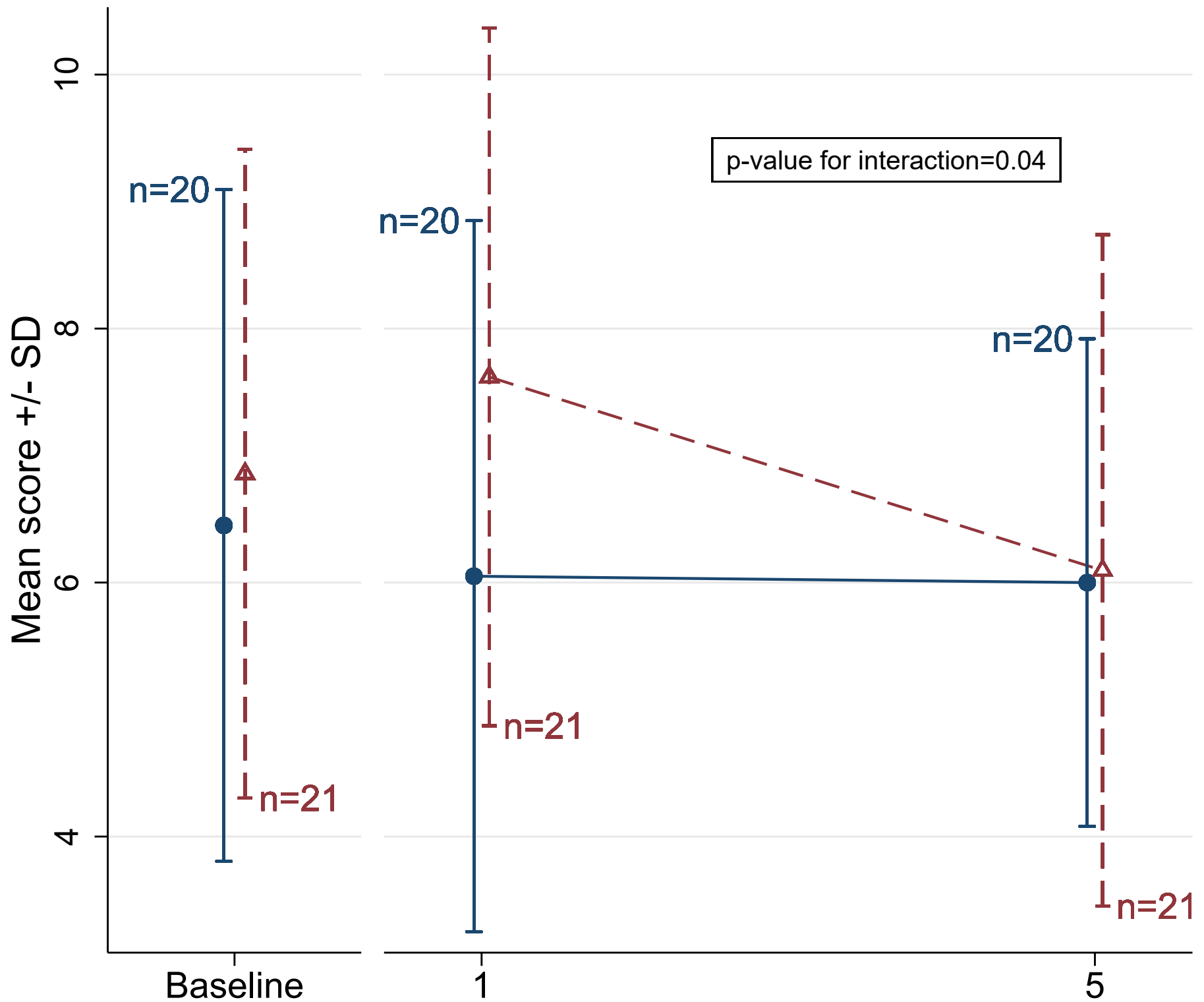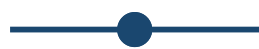

Oral hydrocortisone

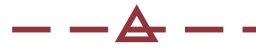

Pulsatile hydrocortisone

Supplement: Supplementary file 3 — Mean score of the Pittsburg Sleep Quality Index (PSQI) at baseline, 1 week and 5 weeks. [file JOIM-295-51-s002.pdf]

# Chalder fatigue scale

## Physical component

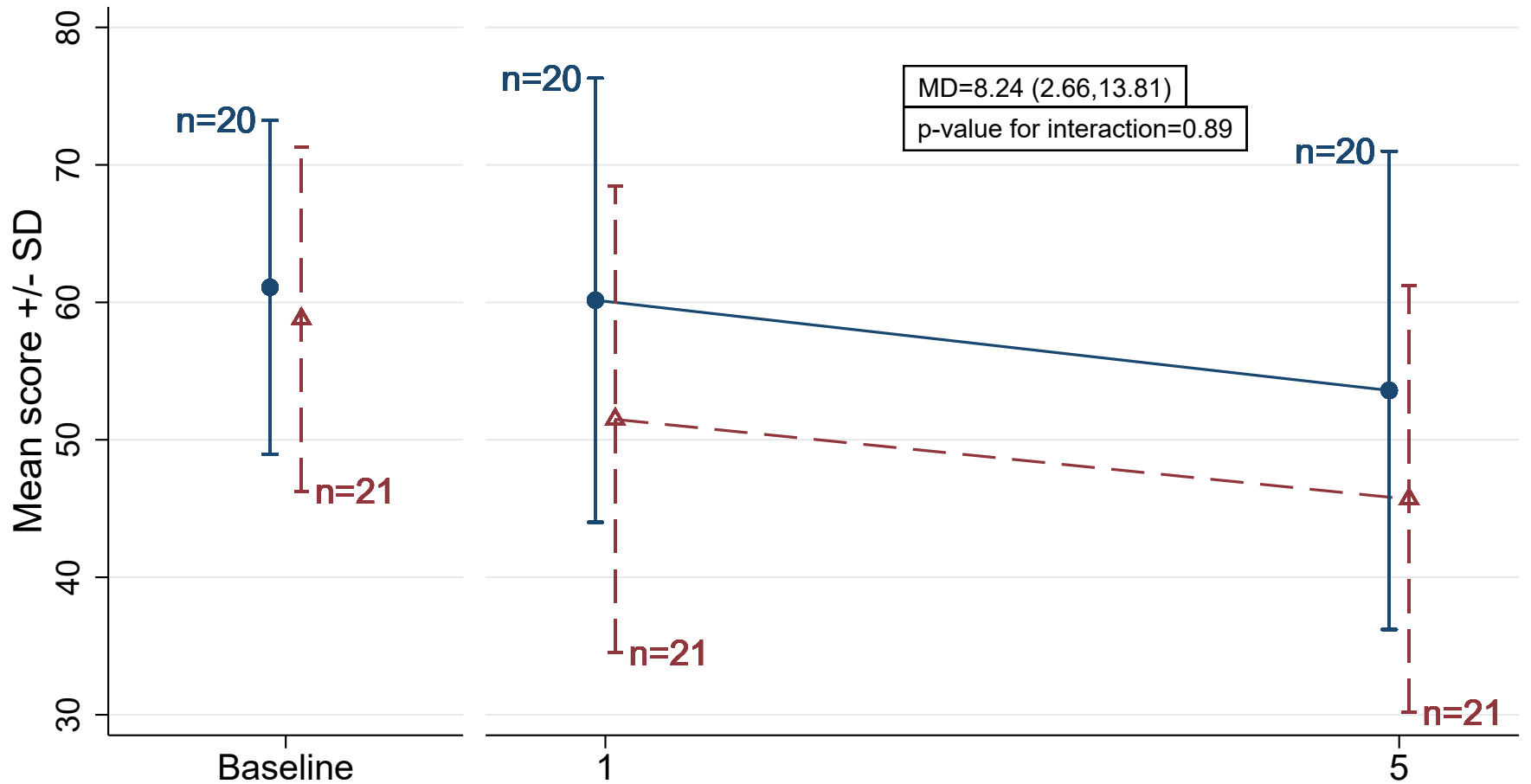

## Mental component

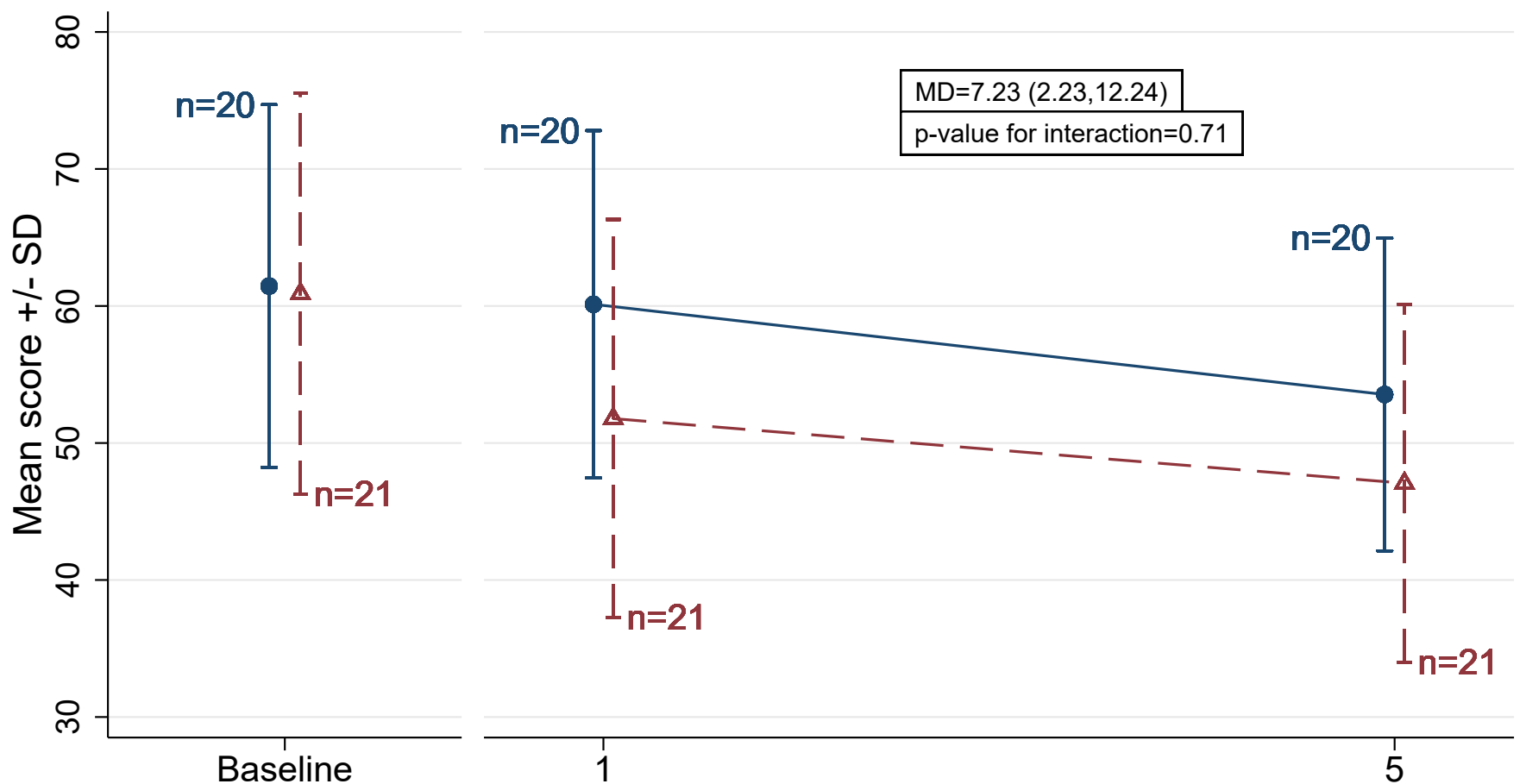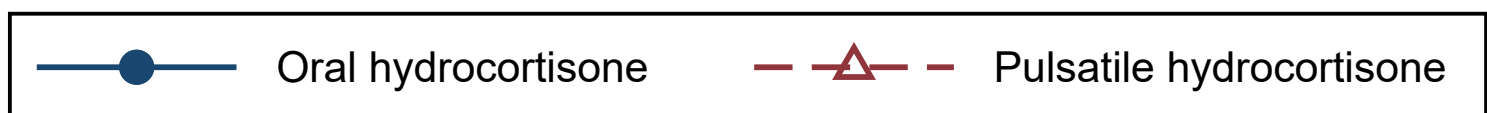

Supplement: Supplementary file 5 — Mean score of the Chalder Fatigue Score at baseline, week 1 and 5. [file JOIM-295-51-s007.pdf]

# Identity-consequence fatigue scale

## Identification of fatigue

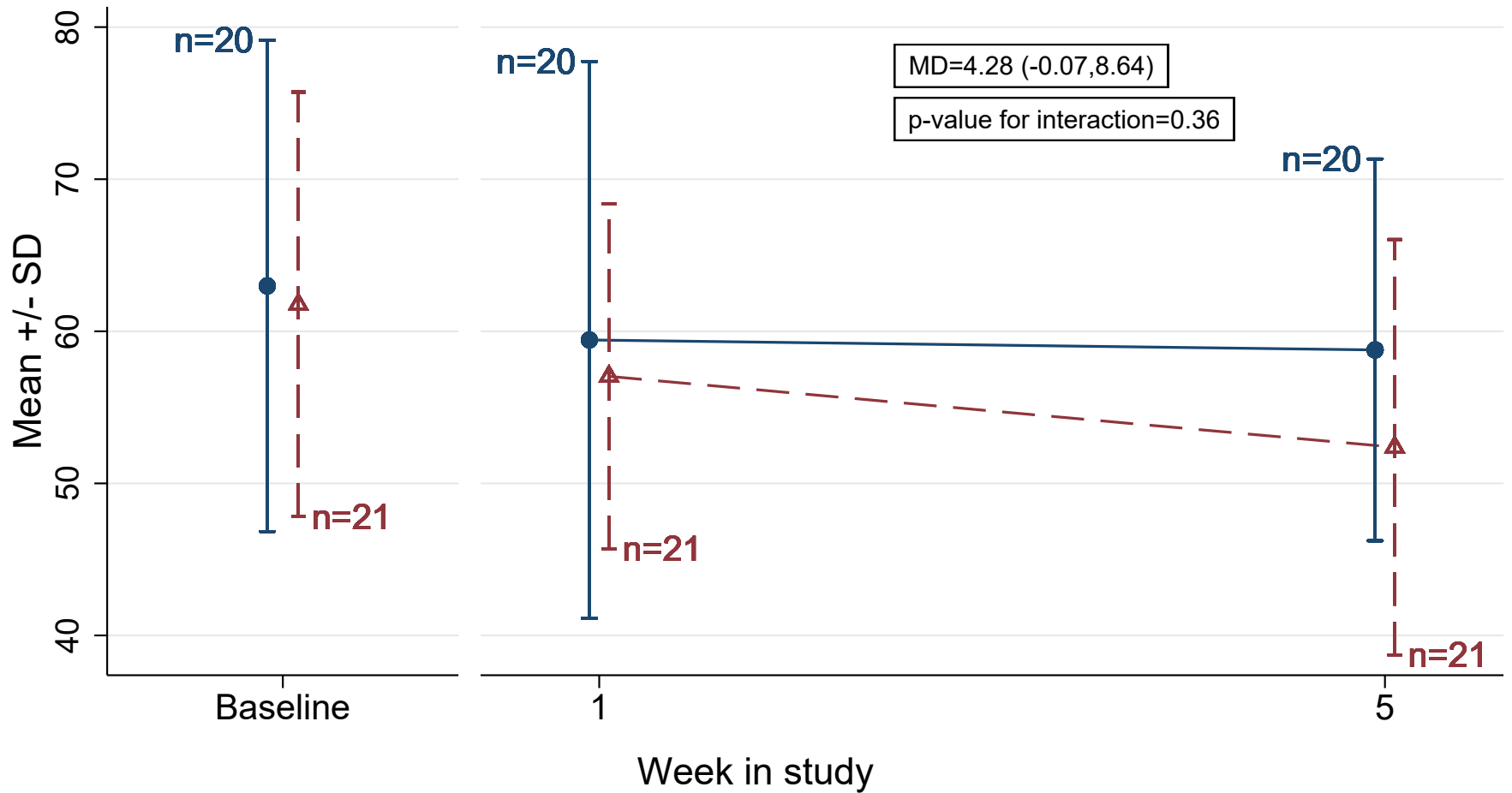

## Consequence of fatigue

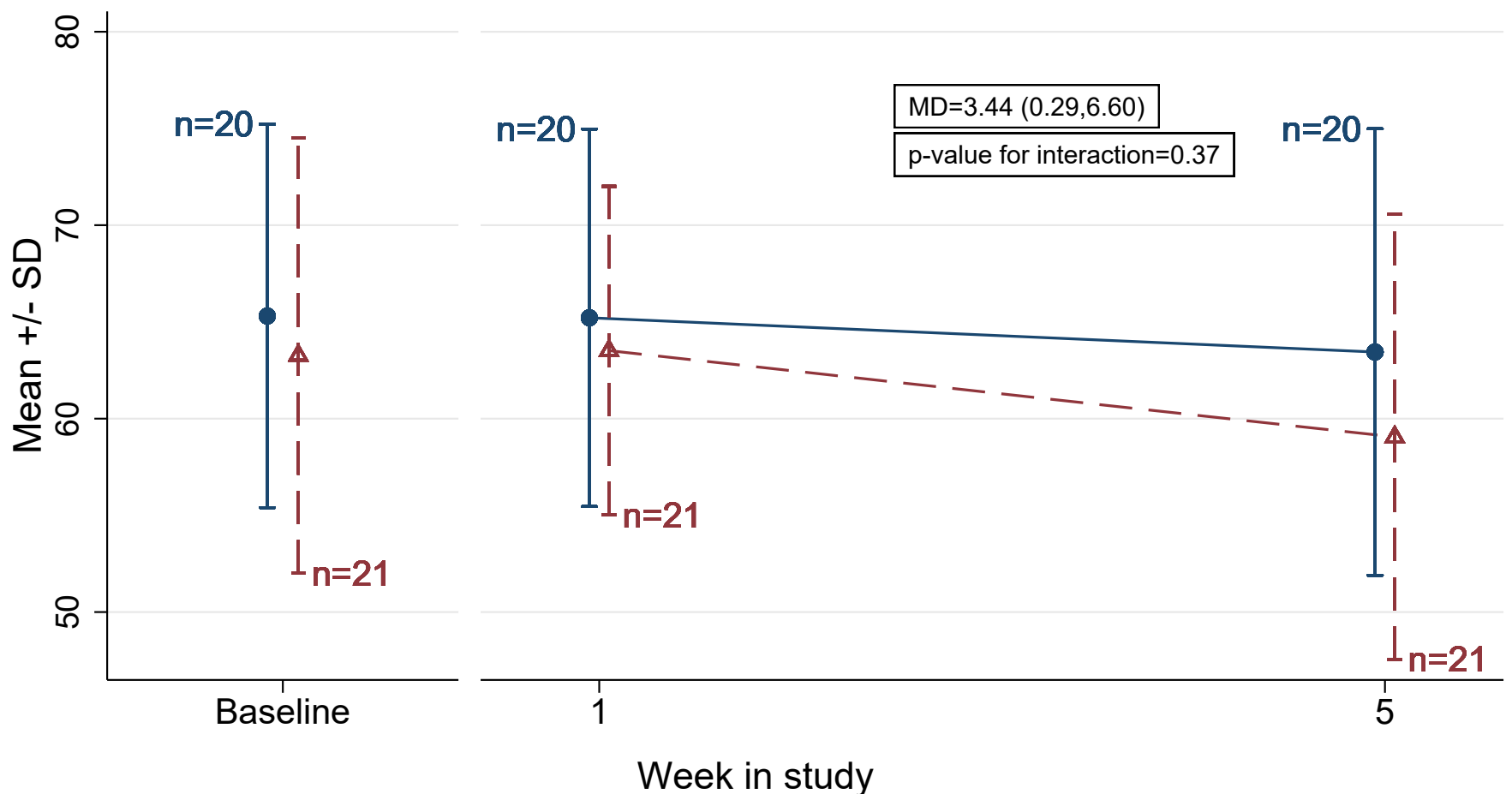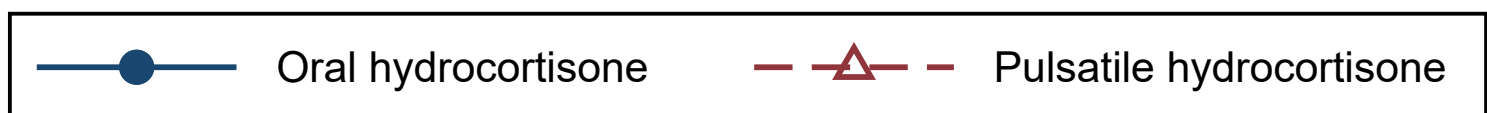

Supplement: Supplementary file 6 — Mean score of the Identity Consequence Fatigue Scale (ICFS) at baseline, week 1 and 5. [file JOIM-295-51-s008.pdf]

# Positive affect negative affect score

## Positive Affect

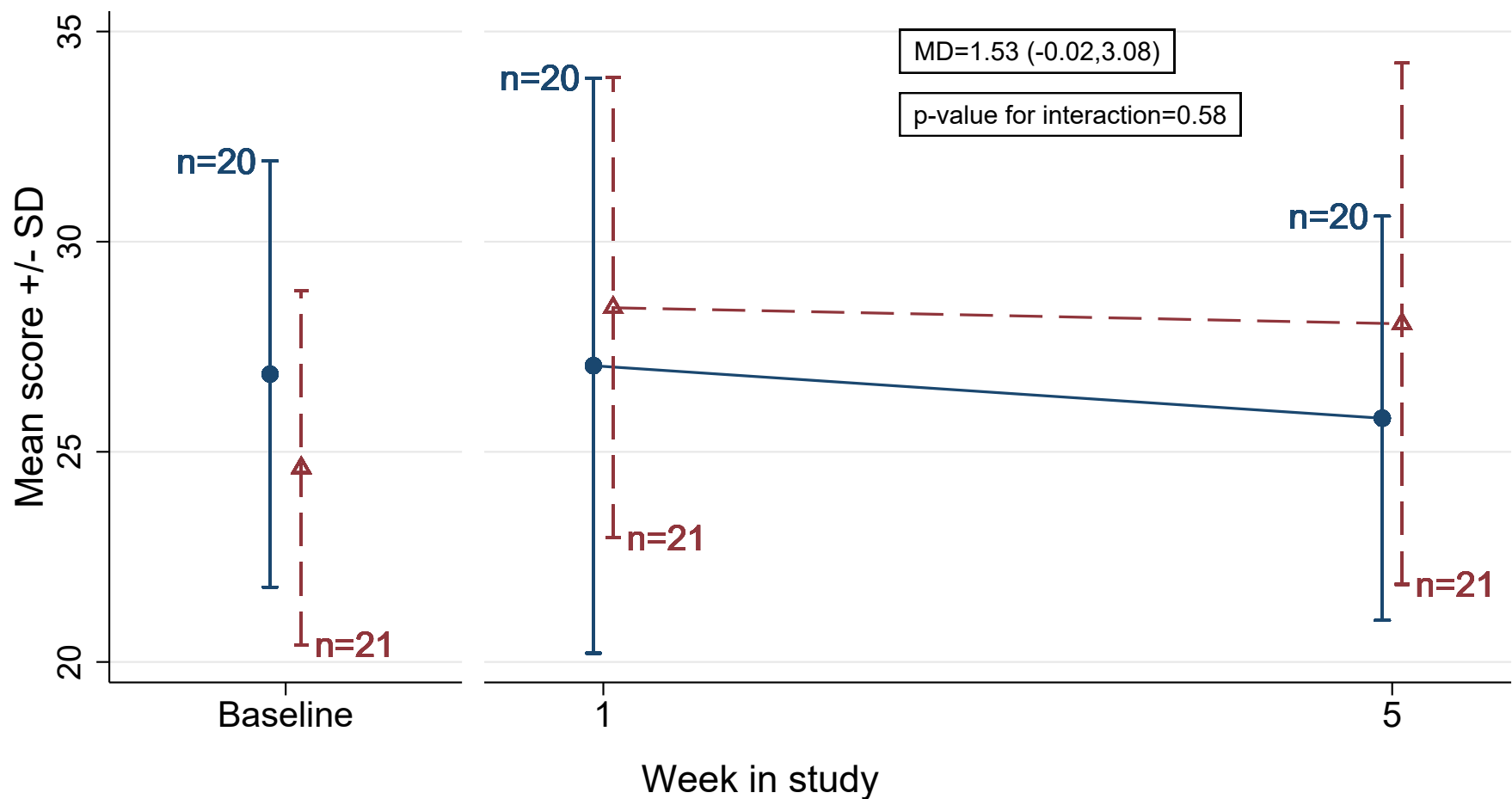

## Negative Affect

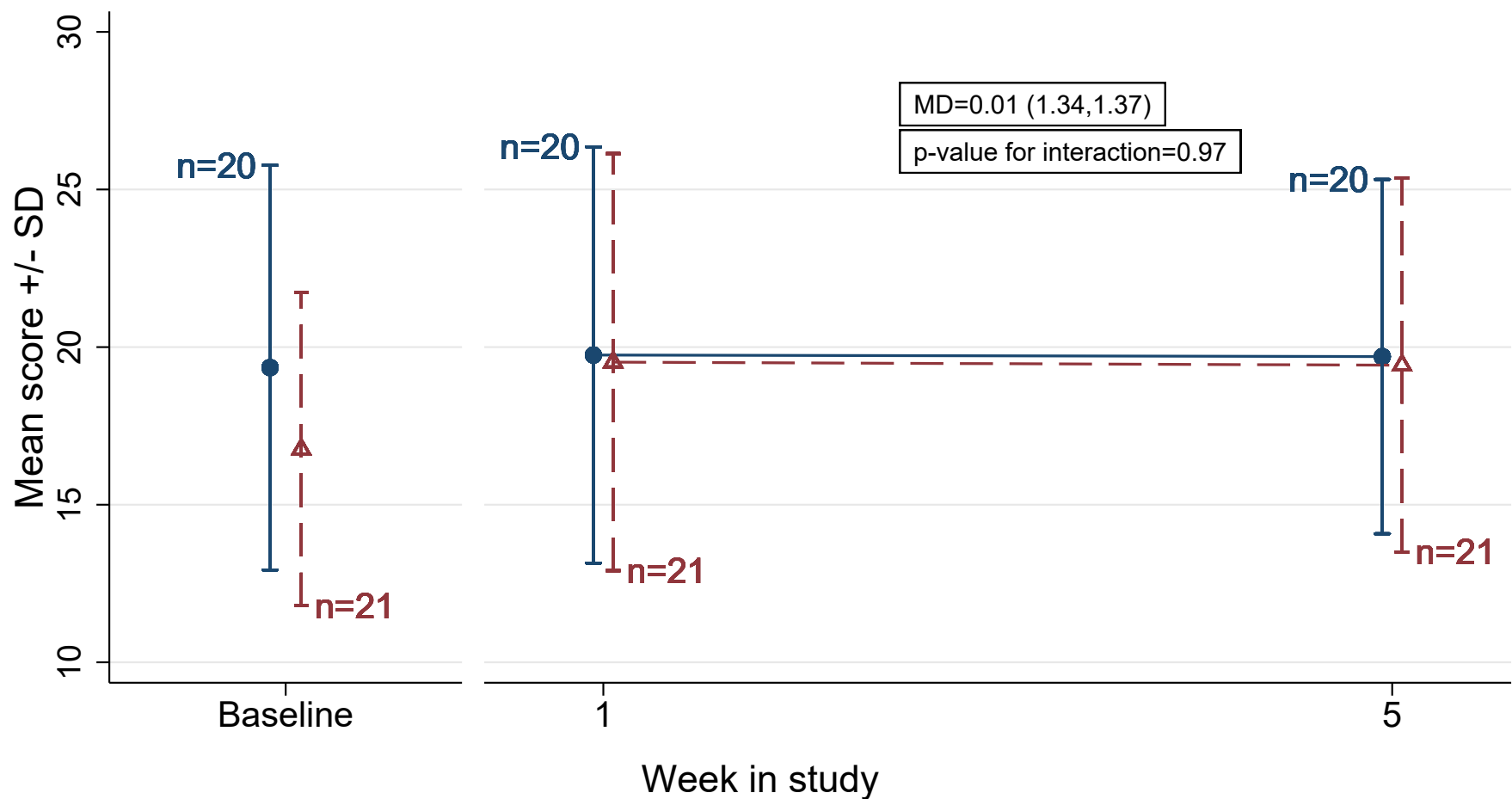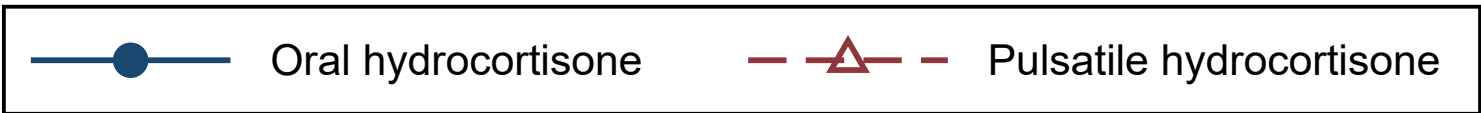

Supplement: Supplementary file 7 — Mean score of the Positive Affect Negative Affect Score (PANAS) at baseline, week 1 and 5. [file JOIM-295-51-s009.pdf]

# Addison's disease Quality of Life

## AddiQol-30 percentage

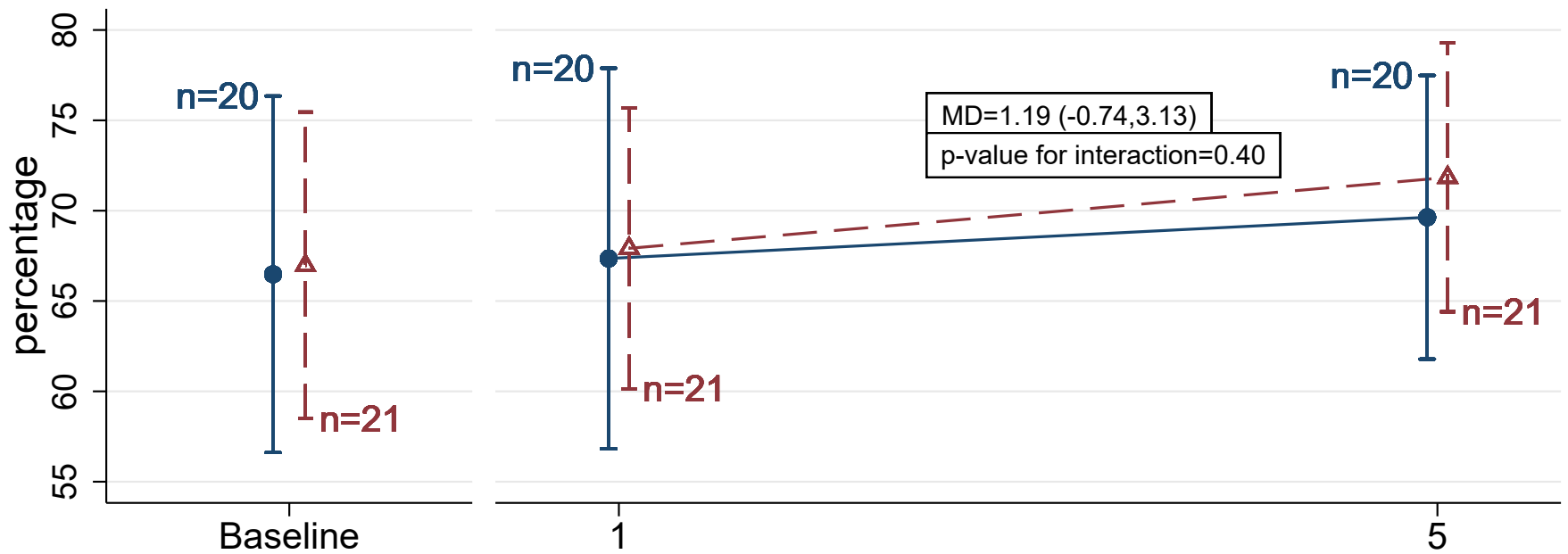

## AddiQol-8 percentage

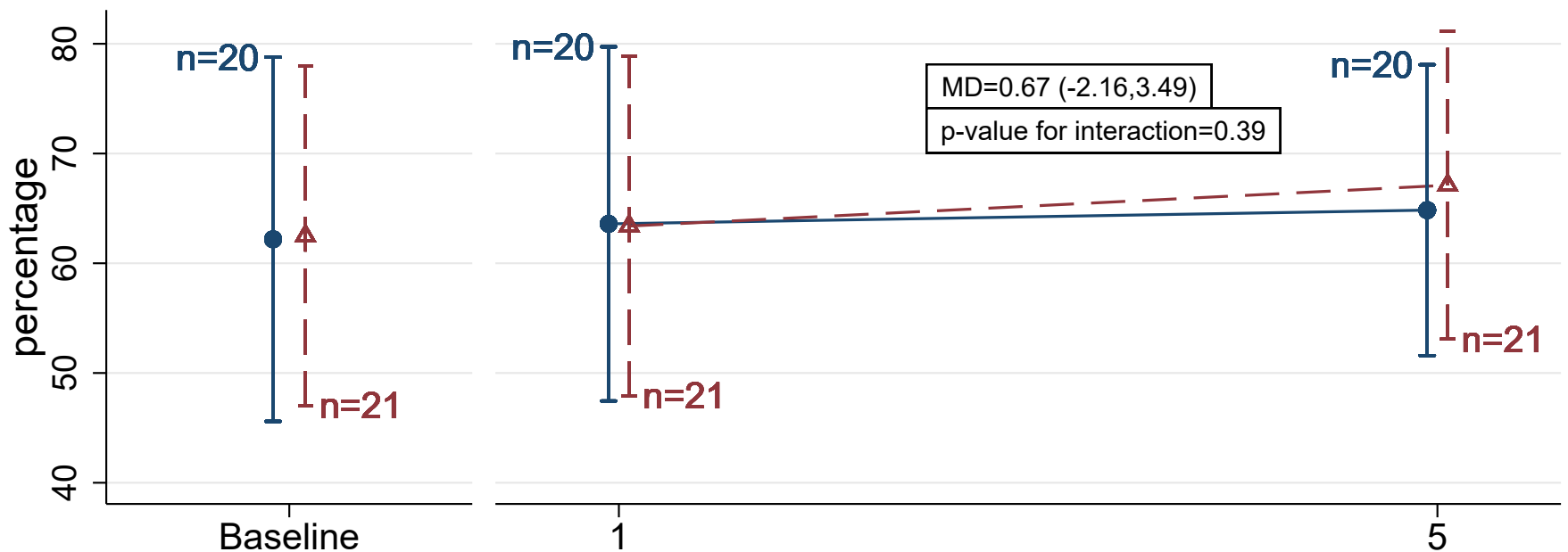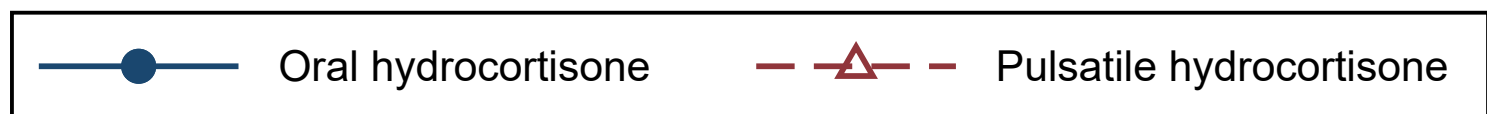

Supplement: Supplementary file 8 — Mean score of the Addison's Disease Quality of Life Scale (AddiQol‐30) at baseline, week 1 and 5. [file JOIM-295-51-s003.pdf]

# Short form (36-item) healthy survey

## Physical functioning

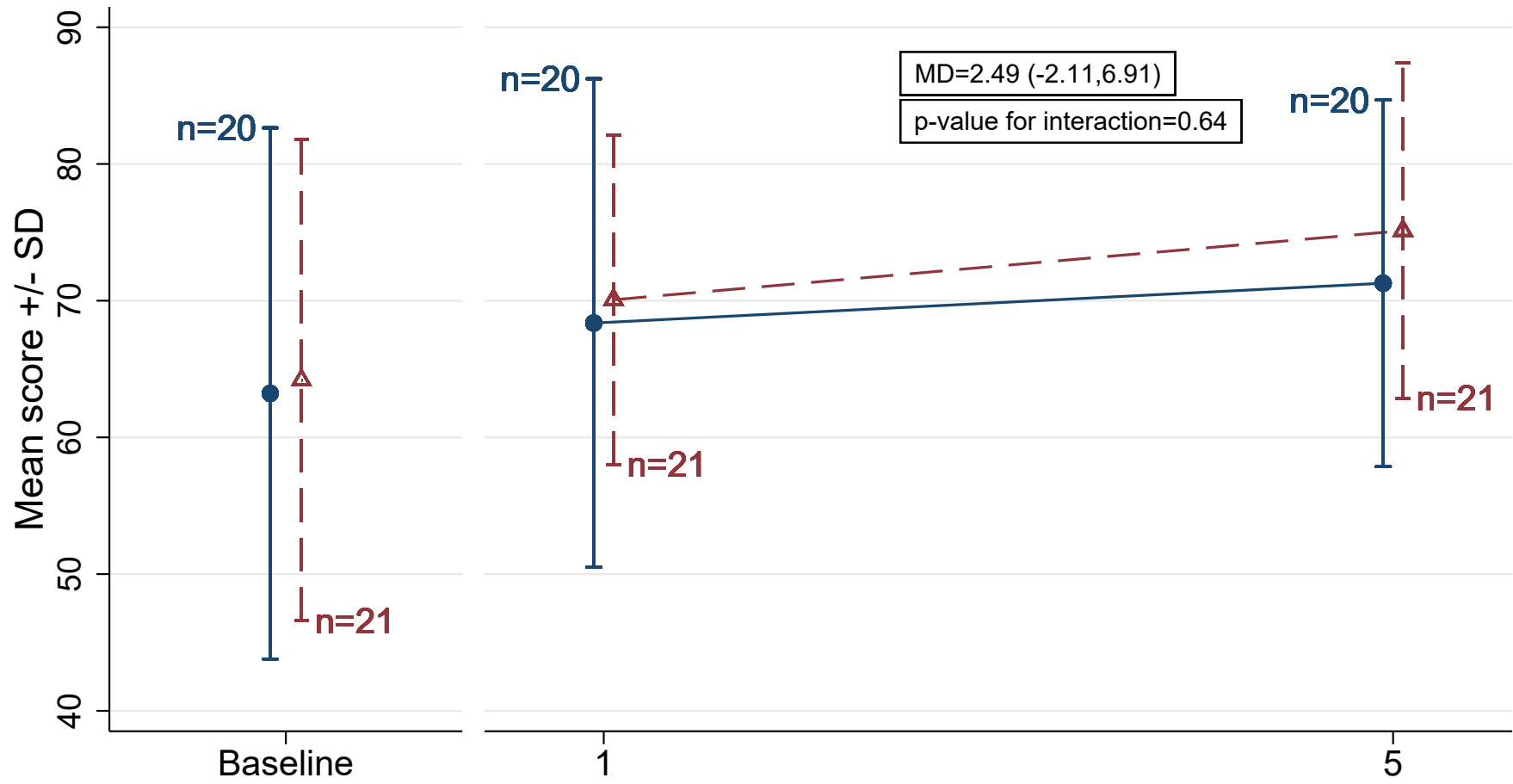

## Mental functioning

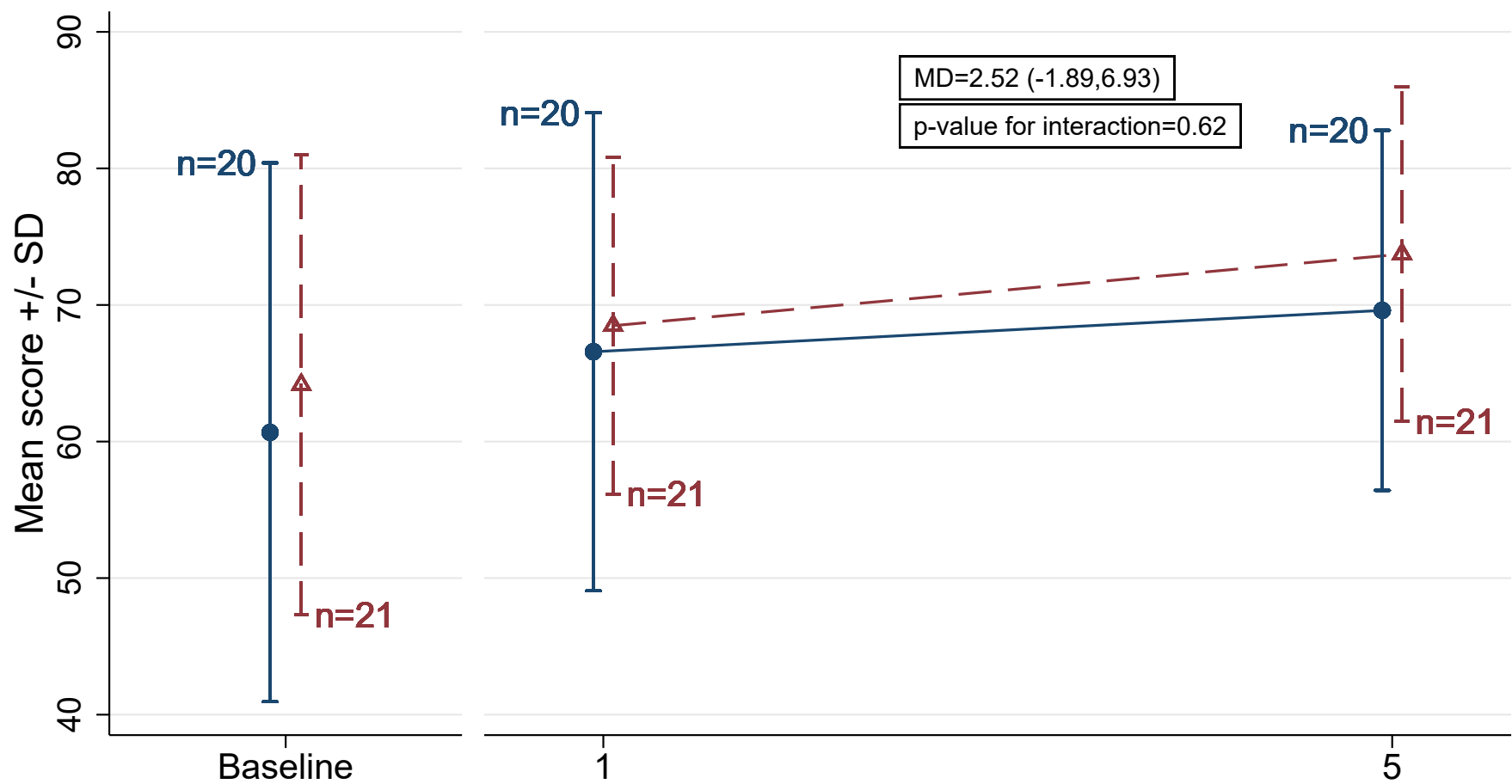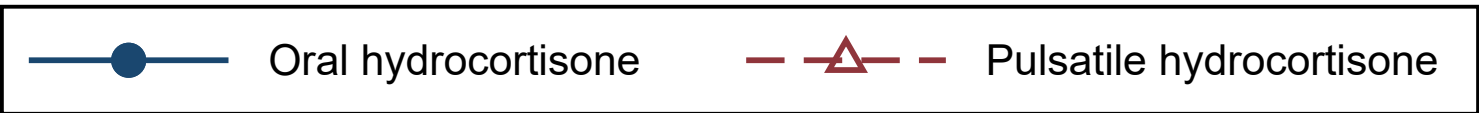

Supplement: Supplementary file 9 — Mean score of the Short Form 36 (SF36) at baseline, week 1 and 5. [file JOIM-295-51-s010.pdf]

# Blood measures

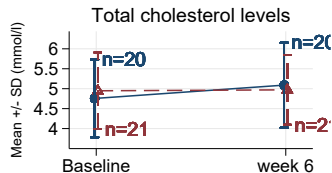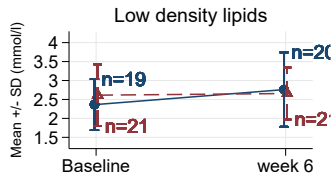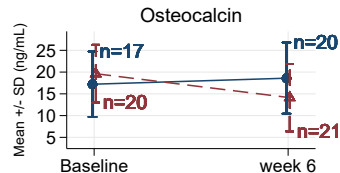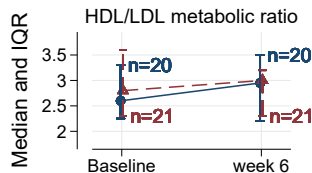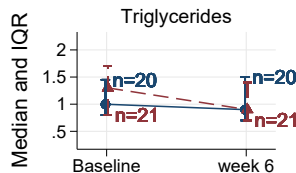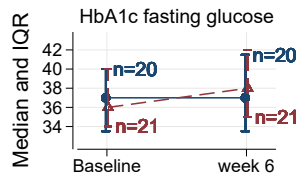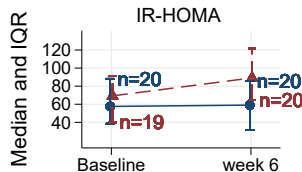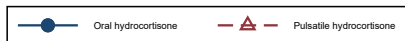

Supplement: Supplementary file 10 — Blood measures of total cholesterol, low density lipids and osteocalcin Blood measures of HDL/LDL metabolic ratio, triglycerides, HbA1c and Insulin resistance. [file JOIM-295-51-s001.pdf]

# Body composition

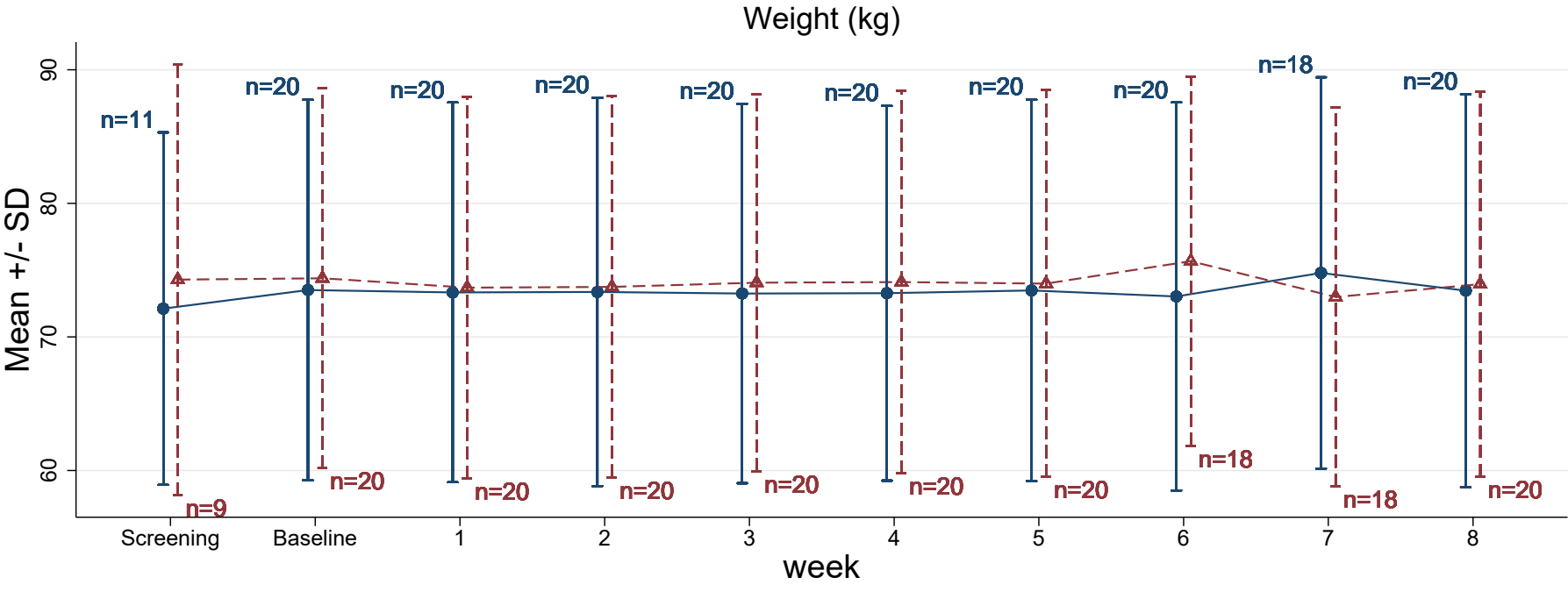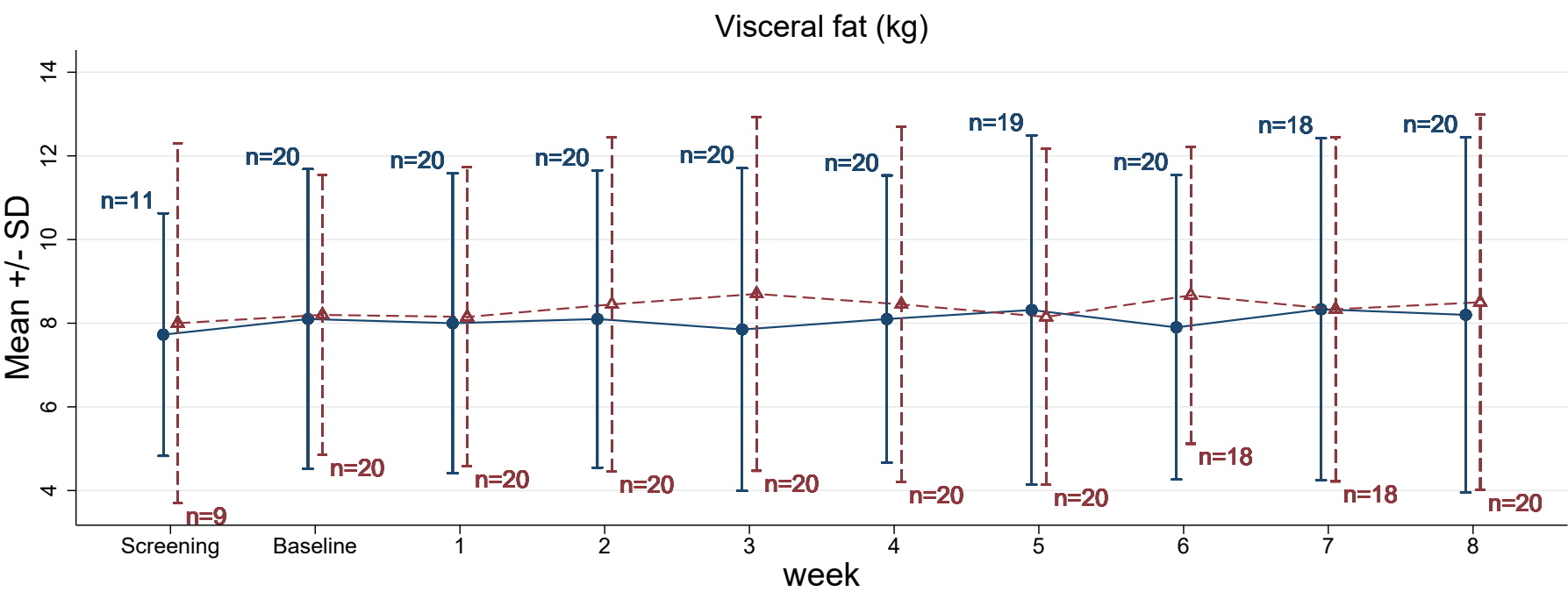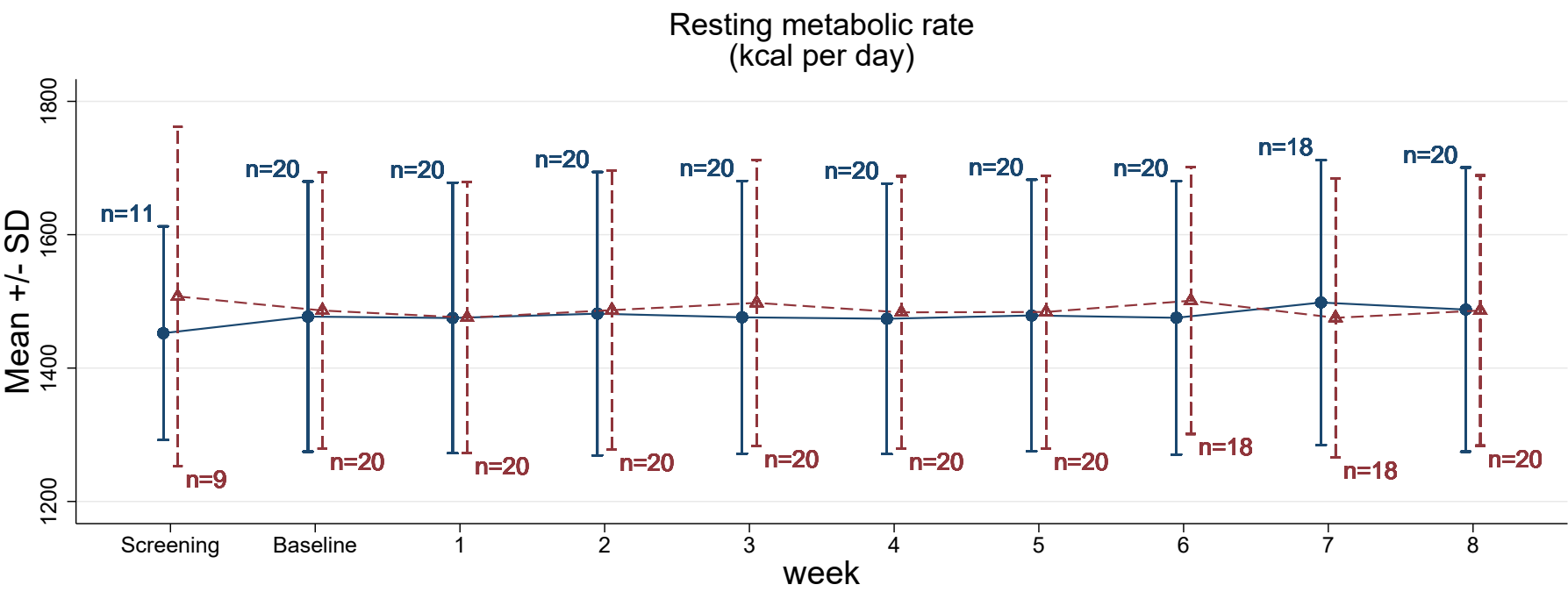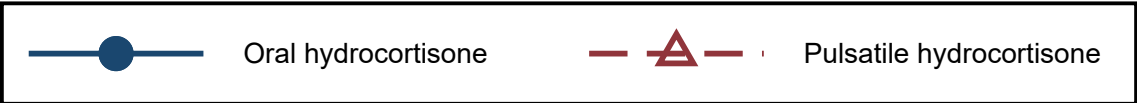

Supplement: Supplementary file 11 — Body composition and resting metabolic rate. [file JOIM-295-51-s006.pdf]
