# Supplementary material for: Ultradian hydrocortisone replacement alters neuronal processing, emotional ambiguity, affect and fatigue in adrenal insufficiency: The PULSES trial
Source: J Intern Med. 2023 Oct 19;295(1):51–67. doi: 10.1111/joim.13721 (PMC10952319; doi:10.1111/joim.13721)
Supplement: Supplementary file 2 — 3 participants' 24‐hour blood profiles for cortisol, ACTH and 17‐OHP (CAH only). Foot note: Participants 1 and 2 were female AD and participant 3 was a male CAH. Red being pulsatile treatment and blue oral. [file JOIM-295-51-s011.pdf]

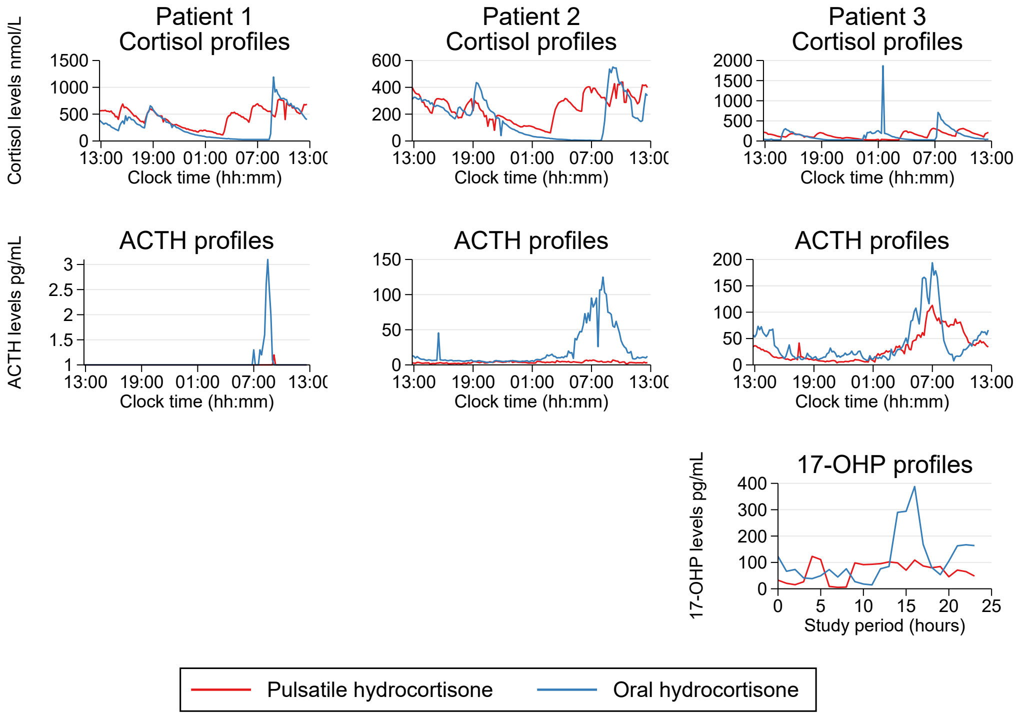
Patient 1
Cortisol profiles
Patient 2
Cortisol profiles
Patient 3
Cortisol profiles
Cortisol levels nmol/L
ACTH profiles
ACTH levels pg/mL
17-OHP profiles
17-OHP levels pg/mL
Clock time (hh:mm)
Study period (hours)
Pulsatile hydrocortisone
Oral hydrocortisone
