# Supplementary material for: Ultradian hydrocortisone replacement alters neuronal processing, emotional ambiguity, affect and fatigue in adrenal insufficiency: The PULSES trial
Source: J Intern Med. 2023 Oct 19;295(1):51–67. doi: 10.1111/joim.13721 (PMC10952319; doi:10.1111/joim.13721)
Supplement: Supplementary file 4 — Mean score of the weekly Leeds Sleep Evaluation Questionnaire. Red open triangle being pulsatile and blue closed circle oral hydrocortisone treatment. [file JOIM-295-51-s004.pdf]

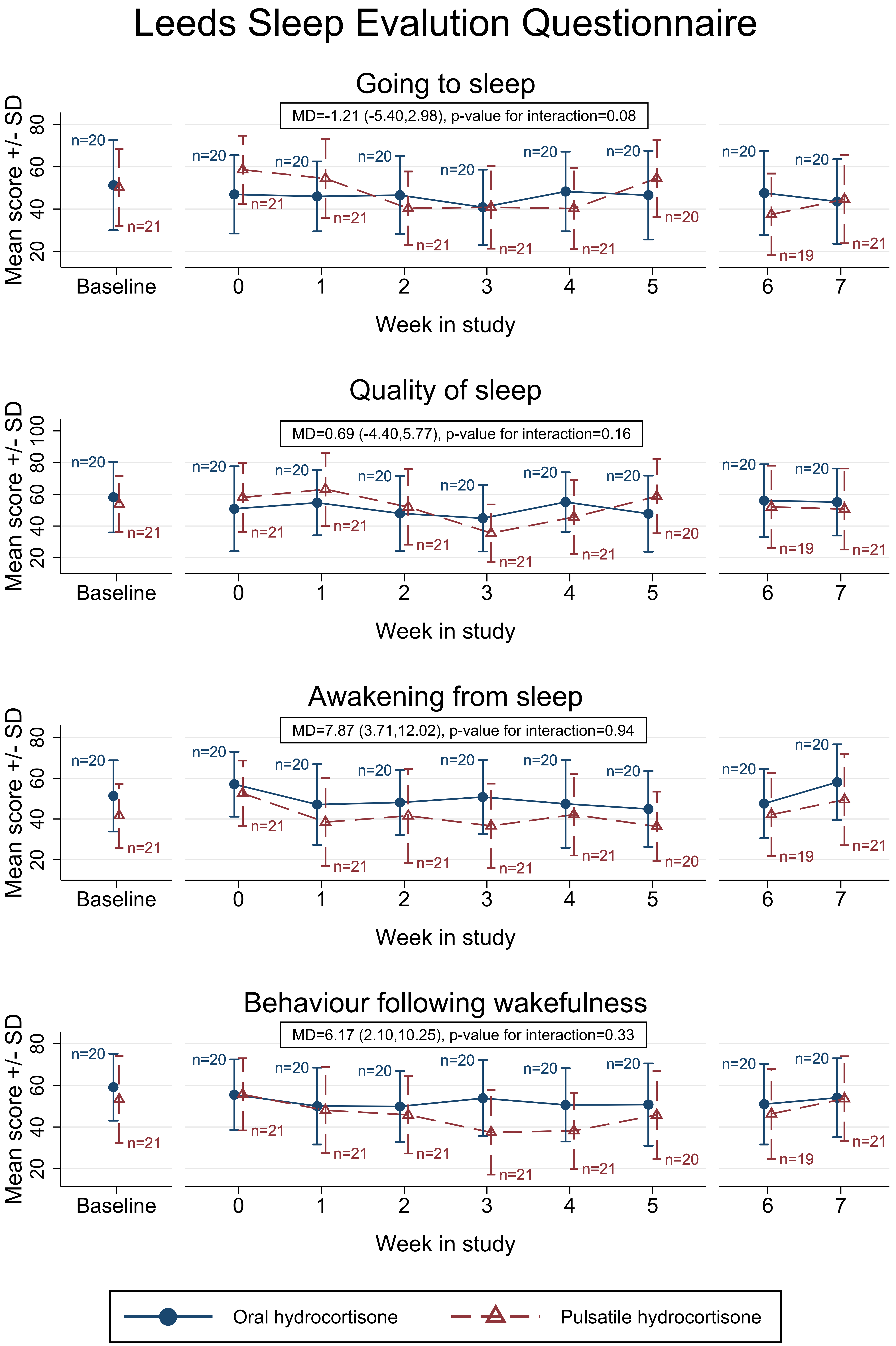
Leeds Sleep Evalution Questionnaire
Going to sleep
MD=-1.21 (-5.40,2.98), p-value for interaction=0.08
Quality of sleep
MD=0.69 (-4.40,5.77), p-value for interaction=0.16
Awakening from sleep
MD=7.87 (3.71,12.02), p-value for interaction=0.94
Behaviour following wakefulness
MD=6.17 (2.10,10.25), p-value for interaction=0.33
Mean score +/- SD
Baseline
0
1
2
3
4
5
6
7
Week in study
n=20
n=21
n=19
Oral hydrocortisone
Pulsatile hydrocortisone
