# Supplementary material for: Ultradian hydrocortisone replacement alters neuronal processing, emotional ambiguity, affect and fatigue in adrenal insufficiency: The PULSES trial
Source: J Intern Med. 2023 Oct 19;295(1):51–67. doi: 10.1111/joim.13721 (PMC10952319; doi:10.1111/joim.13721)
Supplement: Supplementary file 12 — Inclusion and exclusion criteria Example of a total daily dose of 20mg delivered via the subcutaneous pump Schematic diagram of tasks performed during each period of the study ETB (Emotional Test Battery), QoL (Quality of life questionnaires), EMA (Ecological Momentary Assessment), PANAS (Positive and Negative Affect Score), BDI (Beck's Depression Inventory), fMRI (functional Magnetic Resonance imaging) EMA Mood VAS used in morning and evening reports and random prompts. The VAS‐assessment included 10 items: ‘energetic’, ‘alert’, ‘enthusiastic’, ‘happy’, ‘irritable’, ‘sad’, ‘stressed’, ‘unmotivated’,‘upset’ and ‘couch potato’. The latter item was excluded as it has not been validated by other studies. Items were scored on a visual analogue scale (0‐100). Supplementary Table 5 Rotated factor loadings from the constrained two‐factor factor analysis fobrkr the 9 mood items [file JOIM-295-51-s005.docx]

Supplementary Figure 1

**Outline of the key methodological steps for the whole-brain analysis of the functional brain images from the emotional stimulation experiment.**

Similar steps have been followed for the analysis of the functional brain images from the visual stimulation experiment.

The high-resolution, anatomical, T1-weighted images were used for spatially normalizing the low-resolution functional images, and for anatomical localization. They were pre-processed to fit into the co-registration process with the functional images, and standard space. Bias field correction (via a tool of FSL called FAST) has been applied, before removing the non-brain tissue (via a process in SPM called voxel-based morphometry or VBM8).

The functional image pre-processing steps consisted of (i) brain intensity normalization, (ii) 3D motion correction (via a tool in FSL called MCFLIRT), (iii) B0 unwarping with assistance from the B0 fieldmap images (which have been also processed in FSL), (iv) brain extraction (via a tool in FSL called BET), (v) spatial smoothing, (vi) temporal high pass filtering, and (vii) co-registration of functional images with corresponding high-resolution anatomical images and with MNI152 standard space (<https://nist.mni.mcgill.ca/icbm-152lin/>).

For each individual/session fMRI dataset, a regression analysis (with assistance from the FSL tool called FEAT) was performed using a general linear model fitting the temporal evolution of the experiment. A fraction of the temporal derivative of the blurred original waveform was added to the model. Temporal filtering was also applied. The form of the hemodynamic (i.e., BOLD signal) response function convolution method to be applied to the basic waveform was the Gamma variate. Three different effects were modelled (original exploratory variables); visual exposure to (i) fearful human faces, (ii) happy human faces and (iii) sad human faces. (In the case of the visual stimulation experiment, one effect was modelled; the visual exposure to the flashing checkerboard.)

The output of this process produced individual session/subject level maps of activity, indicating which brain regions (across the brain) were showing a hemodynamic response to the emotional face recognition (contrasting the baseline, resting state condition). Three main contrasts were used, representing each separate emotional valence processing (fear, happy, sad). (In the case of the visual stimulation experiment, one contrast was used representing which brain regions across the brain were showing a hemodynamic response to the non-emotional, visual stimulation.)

To find out whether the hemodynamic responses (for each of the 3 emotional valences), across the brain, of the subjects during the emotional stimulation experiment differed notably between the two distinct modes of hydrocortisone replacement therapy, whole-brain, between-group comparisons were carried out using a mixed effects model (which in this case should deliver similar results to a paired t-test). Each analysis (one per emotional valence) produced thresholded z-score brain region clusters, highlighting statistically significant variations in the hemodynamic response between the treatment groups, per contrast used. In all cases, corrections for multiple comparisons were performed at the cluster level using Gaussian random field theory (minimum z > 2.3, cluster p threshold < 0.05). (Similarly, in the case of the visual stimulation experiment, a single analysis produced thresholded z-score brain region clusters, highlighting statistically significant variations in the hemodynamic response between the treatment groups during non-emotional, visual stimulation.)

BOLD: blood oxygen-level dependent, FSL: Software Library for Neuroimaging Analysis developed by the University of Oxford (<https://fsl.fmrib.ox.ac.uk/fsl/fslwiki>), SPM: Statistical Parametric Mapping (University College London) (<https://www.fil.ion.ucl.ac.uk/spm/>)

Supplementary Figure 2

3 participants' 24-hour blood profiles for cortisol, ACTH and 17-OHP (CAH only). Foot note: Participants 1 and 2 were female AD and participant 3 was a male CAH. Red being pulsatile treatment and blue oral.

Supplementary Figure 3

Mean score of the Pittsburg Sleep Quality Index (PSQI) at baseline, 1 week and 5 weeks. Red open triangle being pulsatile and blue closed circle oral hydrocortisone treatment

Supplementary figure 4

Mean score of the weekly Leeds Sleep Evaluation Questionnaire. Red open triangle being pulsatile and blue closed circle oral hydrocortisone treatment.

Supplementary figure 5

Mean score of the Chalder Fatigue Score at baseline, week 1 and 5. Red open triangle being pulsatile and blue closed circle oral hydrocortisone treatment

Supplementary figure 6

Mean score of the Identity Consequence Fatigue Scale (ICFS) at baseline, week 1 and 5. Red open triangle being pulsatile and blue closed circle oral hydrocortisone treatment

Supplementary figure 7

Mean score of the Positive Affect Negative Affect Score (PANAS) at baseline, week 1 and 5. Red open triangle being pulsatile and blue closed circle oral hydrocortisone treatment

Supplementary figure 8

Mean score of the Addison’s Disease Quality of Life Scale (AddiQol-30) at baseline, week 1 and 5. Red open triangle being pulsatile and blue closed circle oral hydrocortisone treatment

Supplementary figure 9

Mean score of the Short Form 36 (SF36) at baseline, week 1 and 5. Red open triangle being pulsatile and blue closed circle oral hydrocortisone treatment

Supplementary Figure 10

Blood measures of total cholesterol, low density lipids and osteocalcin

Blood measures of HDL/LDL metabolic ratio, triglycerides, HbA1c and Insulin resistance. Red open triangle being pulsatile and blue closed circle oral hydrocortisone treatment.

Supplementary figure 11

Body composition and resting metabolic rate. Red open triangle being pulsatile and blue closed circle oral hydrocortisone treatment

| **Inclusion Criteria** | **Exclusion Criteria** |
| --- | --- |
| Male and female patients with confirmed Addison’s disease and CAH | Any significant current cerebral, cardiovascular, respiratory, hepatobiliary, pancreatic disease, renal dysfunction, gastrointestinal emptying or motility disturbances. |
| Aged 18 to 64 years | No current treatment or within the last 3 months of another underlying disease that could necessitate treatment with glucocorticoids |
| Females of childbearing potential must be using a highly effective method of contraception / birth control as defined in ICH (M3) if sexually active | Taking of medications that interfere with cortisol metabolism (antiepileptics, St Johns wart, rifampicin) |
| Right-handed | Diagnosis of Addison’s disease less than 6 months ago |
| Able to give informed consent | Pregnant or lactating women |
|  | Greater than 21 units of alcohol per week |
|  | Taking of any investigational drug within the past two months |
|  | Known allergy to any of the study medications and /or materials used in the pump |
|  | Needle phobia |
|  | Claustrophobia / Contraindication to fMRI scan i.e. metal implant/shrapnel |
|  | Left-handed/significant ambidexterity |
|  | Dyslexia |

Supplementary table 1

Inclusion and exclusion criteria

| **Pulse size**  **(Hydrocortisone dose in mg)** | **Time (24-hour clock)** |
| --- | --- |
| 4 mg | 03.00, 06.00, 09.00 |
| 2.3 mg | 12.00, 15.00, 18.00 |
| 0.5mg | 21.00, 00.00 |

Supplementary table 2

Example of a total daily dose of 20mg delivered via the subcutaneous pump

| Task | -1 weeks | Time 0 | Week | | | | | | |
| --- | --- | --- | --- | --- | --- | --- | --- | --- | --- |
|  |  |  | 1 | 2 | 3 | 4 | 5 | 6 | 7  (wash out) |
| ETB |  |  |  |  |  |  |  |  |  |
| N-back  task |  |  |  |  |  |  |  |  |  |
| QoL |  |  |  |  |  |  |  |  |  |
| Sleep |  |  |  |  |  |  |  |  |  |
| EMA |  |  |  |  |  |  |  |  |  |
| anthropometric |  |  |  |  |  |  |  |  |  |
| PANAS |  |  |  |  |  |  |  |  |  |
| BDI |  |  |  |  |  |  |  |  |  |
| FMRI |  |  |  |  |  |  |  |  |  |
| 24-hour study  (optional) |  |  |  |  |  |  |  |  |  |

Supplementary table 3

Schematic diagram of tasks performed during each period of the study

ETB (Emotional Test Battery), QoL (Quality of life questionnaires), EMA (Ecological Momentary Assessment), PANAS (Positive and Negative Affect Score), BDI (Beck’s Depression Inventory), fMRI (functional Magnetic Resonance imaging)

|  | Header | Feeling right now… |  |  |
| --- | --- | --- | --- | --- |
| VAS_Mood_1 | Question | Alert? | Slider (0-100) | NO!! -- YES!! |
| VAS_Mood_2 | Question | Energetic? | Slider (0-100) | NO!! -- YES!! |
| VAS_Mood_3 | Question | Happy? | Slider (0-100) | NO!! -- YES!! |
| VAS_Mood_4 | Question | Enthusiastic? | Slider (0-100) | NO!! -- YES!! |
| VAS_Mood_5 | Question | Sad? | Slider (0-100) | NO!! -- YES!! |
| VAS_Mood_6 | Question | Upset? | Slider (0-100) | NO!! -- YES!! |
| VAS_Mood_7 | Question | Irritable? | Slider (0-100) | NO!! -- YES!! |
| VAS_Mood_8 | Question | Stressed? | Slider (0-100) | NO!! -- YES!! |
| VAS_Mood_9 | Question | Unmotivated? | Slider (0-100) | NO!! -- YES!! |
| VAS_Mood_10 | Question | Couch potato? | Slider (0-100) | NO!! -- YES!! |

Supplementary table 4

EMA Mood VAS used in morning and evening reports and random prompts. The VAS-assessment included 10 items: ‘energetic’, ‘alert’, ‘enthusiastic’, ‘happy’, ‘irritable’, ‘sad’, ‘stressed’, ‘unmotivated’,’upset’ and ‘couch potato’. The latter item was excluded as it has not been validated by other studies. Items were scored on a visual analogue scale (0-100).

| **Item** | **Communality** | **Factor 1 Positive affect** | **Factor 2 Negative affect** |
| --- | --- | --- | --- |
| energetic | 0.84 | **0.91** | -0.09 |
| enthusiastic | 0.82 | **0.89** | -0.14 |
| unmotivated | 0.72 | **-0.84** | 0.04 |
| alert | 0.72 | **0.84** | -0.09 |
| upset | 0.84 | 0.00 | **0.92** |
| sad | 0.83 | -0.02 | **0.91** |
| stressed | 0.68 | -0.34 | **0.75** |
| happy | 0.68 | 0.56 | -0.57 |
| irritable | 0.47 | -0.46 | 0.51 |
| **Explained variance** |  | **41.0%** | **31.8%** |

Supplementary Table 5 Rotated factor loadings from the constrained two-factor factor analysis for the 9 mood items

Exploratory factor analysis was performed using principal component factor analysis (PCA) to reduce the 9 VAS mood items (from morning, random and evening prompts) to a lower number of variables and to identify empirically related groups. A parallel analysis using a combination of a scree plot and the Kaiser criteria was conducted for factor retaining decision. Moreover, the following criteria were considered for excluding items: (a) loadings lower than 0.40; (b) similar loadings in two or more factors (< 0.10) and calculated the Kaiser-Meyer-Olkin [30]. However, items that had loadings over 0.40 on more than one factor, were excluded from selection of items for the final factor score [31]. Index variables were created depending on the number of factors via an optimally weighted linear combination of the items using item loadings and retention criteria exposed above. The index scores 𝑣 were then rescaled to 𝑤 on the VAS scale using the following transformation: 𝑤=max𝑉𝐴𝑆 𝑠𝑐𝑎𝑙𝑒−min𝑉𝐴𝑆 𝑠𝑐𝑎𝑙𝑒max𝑖𝑛𝑑𝑒𝑥 𝑠𝑐𝑜𝑟𝑒 −min𝑖𝑛𝑑𝑒𝑥 𝑠𝑐𝑜𝑟𝑒∗(𝑣−max𝑖𝑛𝑑𝑒𝑥 𝑠𝑐𝑜𝑟𝑒)+max𝑉𝐴𝑆 𝑠𝑐𝑎𝑙𝑒. The rescaled index scores were modelled using a three-level linear mixed models adjusting for treatment (pulsatile vs oral hydrocortisone administration), with study design variables fitted as fixed effects, and particpant and day variables fitted as random effects.

The Kaiser-Meyer-Olkin test of sampling adequacy (KMO) was 0.819 suggesting a very large degree of nonunique covariance among the items [32]. Similarly, Bartlett’s test of sphericity confirmed significant correlation among at least some of the items measured in the EMA (χ2 = 66022; p < 0.001). The item pool used in the factor analysis was suitable for principal component factor analysis.

The initial EFA indicated two factors with eigenvalues of above 1, and the parallel analysis also recommended considering two factors. Subsequently, a 2-factor solution was chosen and explained 72.8% of the variance. Based on the content of the items that were selected

for each factor, the structure of the new scale reflects the originally intended positive affect (PA) and negative affect (NA).

The positive affect was composed of items related to “I felt energetic”, “I felt enthusiastic”, “I felt unmotivated” and negative affect was composed of items “I felt upset”, “I felt sad” and “I felt stressed” items. The factors accounted for 41% and 31.8% of the variance respectively. Items pertaining to being happy and being irritable loaded over 0.40 for both factors (cross-loading) and therefore were not included in the derived factors.

|  |  | **Oral hydrocortisone period (n=20)** | | | **Pulsatile hydrocortisone (n=20)** | | | **Effect** | **p-value** |
| --- | --- | --- | --- | --- | --- | --- | --- | --- | --- |
|  |  | median | IQR | median | | IQR | (95 % CI) | |  |
| Positive affect index score | Baseline week | 60 | (45.9, 76.9) | 57 | | (39.5, 71.3) |  | |  |
|  | During week 1 | 58 | (42.1, 74.3) | 57 | | (41.0, 73.4) | -1.70 (-3.22, -0.31) | | 0.03 |
|  | During week 2 | 60 | (45.1, 75.7) | 59 | | (41.4, 72.0) | -0.44 (-1.64, 0.76) | | 0.47 |
|  | During week 3 | 60 | (41.0, 74.6) | 62 | | (44.9, 74.6) | 0.82 (-0.14, 1.79) | | 0.09 |
|  | During week 4 | 57 | (42.0, 73.9) | 63 | | (50.1, 79.6) | 2.09 (1.19, 2.98) | | <0.001 |
|  | During week 5 | 57 | (41.4, 75.1) | 63 | | (50.0, 79.3) | 3.53 (2.47, 4.59) | | <0.001 |
|  | During week 6 | 58 | (41.7, 76.2) | 68 | | (51.9, 81.3) | 4.61 (3.32, 5.91) | | <0.001 |
| Interaction treatment*time effect[^[1]^](https://ukc-word-edit.officeapps.live.com/we/wordeditorframe.aspx?ui=en%2DUS&rs=en%2DUS&wopisrc=https%3A%2F%2Fuob-my.sharepoint.com%2Fpersonal%2Fnsgmr_bristol_ac_uk%2F_vti_bin%2Fwopi.ashx%2Ffiles%2F75101c3bb5c54392a036bd50cc21e67e&wdenableroaming=1&mscc=1&wdodb=1&hid=16FF21A0-7060-3000-BC24-4A9724E14DFD&wdorigin=Other&jsapi=1&jsapiver=v1&newsession=1&corrid=cbec3556-8e6f-4fd6-8cc6-f0b86e315089&usid=cbec3556-8e6f-4fd6-8cc6-f0b86e315089&sftc=1&mtf=1&sfp=1&instantedit=1&wopicomplete=1&wdredirectionreason=Unified_SingleFlush&rct=Medium&ctp=LeastProtected#_ftn1) | |  |  |  | |  |  | | <0.001 |
| Negative affect index score | Baseline week | 14 | (3.8, 29.2) | 11 | | (0.0, 23.2) |  | |  |
|  | During week 1 | 13 | (0.7, 32.5) | 12 | | (0.0, 24.3) |  | |  |
|  | During week 2 | 11 | (0.0, 29.3) | 14 | | (0.6, 24.2) |  | |  |
|  | During week 3 | 17 | (0.0, 31.4) | 14 | | (0.0, 24.8) |  | |  |
|  | During week 4 | 16 | (0.4, 32.9) | 15 | | (0.0, 25.5) |  | |  |
|  | During week 5 | 14 | (0.0, 29.6) | 14 | | (0.0, 24.5) |  | |  |
|  | During week 6 | 11 | (0.0, 26.5) | 11 | | (0.0, 23.3) |  | |  |
| Interaction treatment*time effect^1^ | |  |  |  | |  |  | | 0.17 |
| Overall treatment effect | |  |  |  | |  | -2.06 (-2.58, -1.54) | | <0.001 |
| ^1^ Time is modelled in days | |  |  |  | |  |  | |  |

Supplementary Table 6 – Ecological Momentary Assessment (EMA) visual analogue scale (VAS) for positive and negative affect.

|  |  | Oral hydrocortisone period (n=20) | | Pulsatile hydrocortisone (n=20) | | Effect | p-value |
| --- | --- | --- | --- | --- | --- | --- | --- |
|  |  | median (IQR) | | median (IQR) | | (95 % CI) |  |
| ICFS | |  |  |  |  |  |  |
| Feelings of fatigue | Baseline | 66 | (48.0, 80.0) | 72 | (52.0, 80.0) |  |  |
|  | During week 1 | 60 | (44.0, 76.0) | 68 | (48.0, 80.0) | 0.80 (-2.49, 4.10) | 0.63 |
|  | During week 2 | 56 | (44.0, 76.0) | 60 | (48.0, 76.0) | -0.28 (-2.90, 2.35) | 0.84 |
|  | During week 3 | 60 | (40.0, 76.0) | 56 | (40.0, 72.0) | -1.36 (-3.51, 0.79) | 0.22 |
|  | During week 4 | 62 | (44.0, 76.0) | 52 | (40.0, 72.0) | -2.44 (-4.47, -0.41) | 0.02 |
|  | During week 5 | 60 | (40.0, 80.0) | 56 | (40.0, 72.0) | -3.52 (-5.83, -1.21) | 0.003 |
|  | During week 6 | 56 | (40.0, 80.0) | 56 | (40.0, 68.0) | -4.60 (-7.49, -1.71) | 0.002 |
| Interaction treatment*time effect | | | | | |  | 0.02 |
| Feelings of vigour | Baseline | 75 | (60.0, 80.0) | 75 | (65.0, 80.0) |  |  |
|  | During week 1 | 70 | (60.0, 80.0) | 75 | (65.0, 80.0) | -0.15 (-3.04, 2.74) | 0.92 |
|  | During week 2 | 65 | (55.0, 80.0) | 70 | (60.0, 80.0) | -1.19 (-3.46, 1.08) | 0.31 |
|  | During week 3 | 70 | (55.0, 80.0) | 65 | (55.0, 80.0) | -2.22 (-4.05, -0.39) | 0.02 |
|  | During week 4 | 70 | (60.0, 80.0) | 65 | (50.0, 80.0) | -3.26 (-4.98, -1.55) | <0.001 |
|  | During week 5 | 70 | (60.0, 80.0) | 65 | (55.0, 75.0) | -4.30 (-6.29, -2.31) | <0.001 |
|  | During week 6 | 70 | (60.0, 80.0) | 65 | (50.0, 75.0) | -5.34 (-7.86, -2.82) | <0.001 |
| Interaction treatment*time effect | | | | | |  | 0.02 |
| Impact on concentration | Baseline | 68 | (56.0, 72.0) | 68 | (56.0, 72.0) |  |  |
|  | During week 1 | 64 | (48.0, 72.0) | 64 | (48.0, 72.0) |  |  |
|  | During week 2 | 64 | (48.0, 72.0) | 64 | (48.0, 72.0) |  |  |
|  | During week 3 | 60 | (48.0, 72.0) | 56 | (48.0, 72.0) |  |  |
|  | During week 4 | 60 | (48.0, 72.0) | 56 | (48.0, 68.0) |  |  |
|  | During week 5 | 60 | (48.0, 72.0) | 60 | (48.0, 68.0) |  |  |
|  | During week 6 | 60 | (48.0, 72.0) | 56 | (48.0, 68.0) |  |  |
| Interaction treatment*time effect | | | | | |  | 0.45 |
| Overall treatment effect | | | | | | -1.44 (-2.74, -0.15) | 0.03 |
| Impact on energy | Baseline | 65 | (50.0, 75.0) | 65 | (55.0, 80.0) |  |  |
|  | During week 1 | 60 | (45.0, 75.0) | 65 | (55.0, 75.0) | 1.76 (-1.02, 4.55) | 0.21 |
|  | During week 2 | 55 | (45.0, 70.0) | 60 | (50.0, 70.0) | 0.94 (-1.30, 3.18) | 0.41 |
|  | During week 3 | 60 | (45.0, 75.0) | 60 | (45.0, 75.0) | 0.11 (-1.75, 1.98) | 0.90 |
|  | During week 4 | 60 | (50.0, 75.0) | 60 | (40.0, 75.0) | -0.71 (-2.48, 1.06) | 0.13 |
|  | During week 5 | 60 | (45.0, 75.0) | 60 | (45.0, 75.0) | -1.54 (-3.55, 0.47) | 0.13 |
|  | During week 6 | 60 | (50.0, 70.0) | 55 | (45.0, 70.0) | -2.36 (-4.84, 0.12) | 0.05 |
| Interaction treatment*time effect | | | | | |  | 0.04 |
| Impacts on daily activities | Baseline | 64 | (52.7, 76.4) | 69 | (52.7, 76.4) |  |  |
|  | During week 1 | 71 | (54.5, 78.2) | 65 | (54.5, 76.4) |  |  |
|  | During week 2 | 73 | (60.0, 78.2) | 70 | (60.0, 77.3) |  |  |
|  | During week 3 | 73 | (65.5, 78.2) | 71 | (60.0, 80.0) |  |  |
|  | During week 4 | 72 | (65.5, 76.4) | 67 | (58.2, 80.0) |  |  |
|  | During week 5 | 69 | (60.0, 76.4) | 70 | (61.8, 80.0) |  |  |
|  | During week 6 | 73 | (65.5, 76.4) | 73 | (65.5, 80.0) |  |  |
| Interaction treatment*time effect | | | | | |  | 0.33 |
| Overall treatment effect | | | | | | 2.29 (0.83,3.75) | 0.002 |

Supplementary Table 7 ICFS EMA analysis - Five subscales were created according to the validated subscales used to score the ICFS in each participant group [15]. The subscale “vigour” was created from the following questionnaire items: “I feel energetic”, “I feel refreshed”, “I feel vigorous” and “I feel lively”. “Fatigue” was created from the following questionnaire items: “I feel drained”, “I quickly become tired”, “I feel worn out”, “My body feels heavy all over” and “I feel physically tired”. “Impact on concentration” was created from the following questionnaire items: “I am unable to concentrate”, “Right now tiredness means I have trouble paying attention”, “Right now tiredness means I am forgetful”, “Right now tiredness means my thoughts wander” and “Right now tiredness means I make more mistakes”. “Impact on motivation” was created from the following questionnaire items: “I have energy to do lots of things” (reverse-scored), “I have to restrict how much I do” and “I can’t get motivated/ do regular activities”. “Activity” was created from the following questionnaire items: “Right now I have energy to read a newspaper, book or watch TV”, “Right now I have energy to bath or wash”, “Right now I have energy to dress”, “Right now I have energy to do housework”, “Right now I have energy to cook”, “Right now I have energy to work”, “Right now I have energy to visit/ socialize with family/ friends”, “Right now I have energy to engage in leisure/ recreational activities”, “Right now I have energy to shop/ do errands”,  “Right now I have energy to walk” and “Right now I have energy to exercise (not walking)”.

Supplementary references

34. Tabachnick, B. G., & Fidell, L. S. (2007). Using Multivariate Statistics (5th ed.). New York: Allyn and Bacon.

35. Ferguson, E., & Cox, T. (1993). Exploratory factor analysis: A user’s guide. International Journal of Selection and Assessment, 1, 84-94.

36. Kaiser, H. F. (1974). An index of factorial simplicity. Psychometrika, 39, 31–36.
